# Phylo SI: a new genome-wide approach for prokaryotic phylogeny

**DOI:** 10.1093/nar/gkt1138

**Published:** 2013-11-15

**Authors:** Anton Shifman, Noga Ninyo, Uri Gophna, Sagi Snir

**Affiliations:** ^1^Department of Evolutionary & Environmental Biology, University of Haifa, Haifa 31905 Israel, ^2^Department of Molecular Microbiology and Biotechnology Tel Aviv University, Tel Aviv 69978, Israel and ^3^National Evolutionary Synthesis Center, 2024 W. Main Street A200, Durham, NC 27705, USA

## Abstract

The evolutionary history of all life forms is usually represented as a vertical tree-like process. In prokaryotes, however, the vertical signal is partly obscured by the massive influence of horizontal gene transfer (HGT). The HGT creates widespread discordance between evolutionary histories of different genes as genomes become mosaics of gene histories. Thus, the Tree of Life (TOL) has been questioned as an appropriate representation of the evolution of prokaryotes. Nevertheless a common hypothesis is that prokaryotic evolution is primarily tree-like, and a routine effort is made to place new isolates in their appropriate location in the TOL. Moreover, it appears desirable to exploit non–tree-like evolutionary processes for the task of microbial classification. In this work, we present a novel technique that builds on the straightforward observation that gene order conservation (‘synteny’) decreases in time as a result of gene mobility. This is particularly true in prokaryotes, mainly due to HGT. Using a ‘synteny index’ (SI) that measures the average synteny between a pair of genomes, we developed the phylogenetic reconstruction tool ‘Phylo SI’. Phylo SI offers several attractive properties such as easy bootstrapping, high sensitivity in cases where phylogenetic signal is weak and computational efficiency. Phylo SI was tested both on simulated data and on two bacterial data sets and compared with two well-established phylogenetic methods. Phylo SI is particularly efficient on short evolutionary distances where synteny footprints remain detectable, whereas the nucleotide substitution signal is too weak for reliable sequence-based phylogenetic reconstruction. The method is publicly available at http://research.haifa.ac.il/ssagi/software/PhyloSI.zip.

## INTRODUCTION

The ever decreasing sequencing costs, and improvements in assembly algorithms and automated annotation have resulted in thousands of bacterial genomes that are being sequenced each year. In fact, cheaper smaller sequencing machines (the so called ‘personal genome sequencers’) are now finding their way to more and more research laboratories and clinics. However, in many cases, to accurately taxonomically place a new isolate remains a serious challenge. Ribosomal RNA genes often do not provide sufficient phylogenetic resolution or show intra-genomic variability, and thus whole-genome data should ideally be used, but deriving taxonomy from these data can be difficult. Selecting multiple house-keeping genes and concatenating them, aligning them with similar concatenations from other bacteria and reconstructing phylogenies requires substantial knowhow, and still has significant drawbacks and cannot always fully resolve the position of the new taxon on the tree (1,2). Ideally, one would harness the whole genomic information and increase the phylogenetic signal, but genomes of bacteria and archaea are characterized by numerous horizontal gene transfer (HGT) (3–6). Thus, different genes in the genome can have different evolutionary histories and conflicting phylogenetic signals, so lumping them together could result in inaccurate organismal phylogenies.

Whether prokaryotic evolution should be portrayed by a vertical single ancestor process or as a network of ancestral relationship, is among the most controversial in microbial evolution (3,7). Views range from one extreme, claiming that, due to HGT, a single tree is far from adequately representing microbial evolution (4,8), to the other extreme that HGT is insignificant in terms of its overall impact on the evolutionary process (1,9,10). Nevertheless, even without taking a side in this dispute, the growing need to classify new genomic data consequently imposes a need to develop new efficient classification techniques. Hence, it would be desirable to harness the non–tree-like evolutionary processes for the goal of classification (11).

We distinguish between ‘sequence (or nucleotide)-based’ and ‘gene-based’ phylogenetic methods. In sequence based methods, orthologous sequences, that is, sequences homologous via speciation (12), from various organisms are used to infer the evolutionary history of the corresponding gene. These methods were the first to be used in molecular systematics thanks to the strong correlation between histories of many evolutionarily conserved genes and the organisms analyzed, mainly animals and plants (13,14). In contrast, the gene-based phylogenetics is a genome-wide approach, where a coarser resolution is used in a broader view. Here, relations between the genomes as sets of genes are exploited to infer similarity (or dissimilarity) between organisms. This work focuses on this family of phylogenetic approaches.

Historically, although the gene-based approaches are more recent than the sequence-based approaches, they were suggested and have been in use for more than two decades. The gene-based family of methods can be further divided into two main subfamilies: gene order–based and gene content (GC; presence/absence)–based techniques. Perhaps the most prominent among the gene order techniques is the genome rearrangement approach that defines the ‘distance’ between genomes as the number of operations (i.e. rearrangement events) required to turn one genome into another. The pioneering studies of Sankoff and colleagues (15,16) were the first to construct phylogenies based on this type of data. However, they were preceded by other studies pointing to the linkage between genome rearrangement events and evolutionary relatedness, starting with the classical work of Dobzhansky and Sturtevant on inversions in Drosophila chromosomes (17) and several others thereafter (18). There has been a wealth of mathematical and biological extensions to the initial model, with more operations and finer algorithms and analysis [see (19–23) among many] including available software (24,25). This gene rearrangement approach assumes a stable set of genes and cannot readily model events of gene gain/loss (26). When organisms liable to the latter type of events are analyzed, genes acquired via HGT are removed and the analysis is done only on the set of genes present in all species (27) (although some new models, e.g. (28), do account for such events but, to the best of our knowledge, no software was produced). The other GC-based approach, the GC approach that is more suitable for the study of prokaryotic evolution, is based on the presence/absence of orthologous genes (29–31). Here the order between the location of the genes on the chromosome is ignored and hence a randomly permuted genome is indistinguishable (zero distance) from the original genome. An advantage of this method over the gene order technique is its speed and hence its ability to analyze larger sets of organisms and genes. Other intermediate gene-based approaches have been proposed between the two extremes of complete order dependent and presence/absence (32–34). In addition, genome-wide sequence-based methods have been developed such as Average Nucleotide Identity (35) or average BLAST scores (36). However, these methods generally did not perform better than gene sequence–based methods, such as those based on the 16S-rRNA (36).

In (26), several of the above approaches were compared for their ability to resolve prokaryotic evolution. The authors concluded that ‘extension of phylogenetic analysis to the genome scale has the potential of uncovering deep evolutionary relationships between prokaryotic lineages’. Further, in (34) it is noted that ‘HGT could significantly affect trees reconstructed using any method of genome composition analysis’. Importantly, a study using gene-order information (37) tracked genome-wide synteny loss between closely related prokaryotes. This approach, conceptually similar to the one we present here, showed that loss of synteny as a result of genome rearrangement events, is very strongly correlated with amino acid distance.

Taking into consideration the developments discussed above, a novel gene-based technique, ‘Phylo SI’, is proposed here that is most efficient for closely related organisms. Phylo SI combines the two existing gene-based approaches, gene order and GC, and aims to trace specific events that are typical of the evolution of prokaryotes with higher sensitivity. The idea relies on the dynamic nature of prokaryotic genomes, with intensive genome mobility, resulting in high rates of HGT. The footprint of this activity in the specific genome architecture, is a patchy phyletic pattern (38) in which a genome contains DNA patches from several different ancestral sources. The proposed technique builds on a new measure that we define as the ‘synteny index’ (SI) between two genomes (species). Gene synteny (39,40) is the conservation of gene order across species along the evolutionary course (It is worth noting that ‘synteny’ in the strictest sense means only that genes are present on the genome; however, we here use the common sense of conserved linkage.). The SI measures how much a gene that is orthologous in the two compared species is in its ‘natural place’, or in other words, shares the same neighborhood in both genomes. During evolution, a genome undergoes events of large scale reorganization, such as gene gain/loss, duplication and translocation, causing a degradation in the synteny among the genomes (41). Although synteny over several genes may not be informative systematically, aggregating synteny data over whole genomes enables a fairly accurate estimation of the evolutionary distance between organisms. Notably, the traditional sequence-based phylogenetic analysis is heavily dependent on identification of orthologous genes among species [e.g. Clusters of Orthologous Groups database (COGs) (42)], correct multiple alignment of the sequences (43) and eventually, accurate phylogenetic reconstruction (44). Our method is independent of most of these tasks (although orthology identification is also a crucial part in this case, inaccuracies may be alleviated by using large sets of gene families). Among the main advantages of the new method, is its easy and efficient implementation, high sensitivity in cases where mutation signal may be too weak for highly conserved genes that are normally used in phylogenetics, lack of model assumptions, aggregation of information across the whole genome and simple implementation of bootstrapping to obtain statistical support for the observed branches. Moreover, the proposed method allows one to trace the evolution of the genome architecture, where species with similar architectures (in terms of synteny and GC) are considered related.

We implemented the proposed method in software and tested it in both simulation and real genomic data analysis. In the simulation study, we compared the method with both GC and gene order techniques, and with an intermediate technique, ‘gene pairs’. A clear advantage was demonstrated over the competing methods, in particular in the analysis of closely related species. In the real genomic data study, the method was meticulously tested on two bacterial data sets. In the first data set, the groupings identified by Phylo SI are in significant agreement with standard phylogenetic approaches such as the highly popular Interactive Tree Of Life (iTOL) tool (1), and the AutoMated PHylogenOmic infeRence Application for large-scale protein phylogenetic analysis (AMPHORA) suit (2) that use a multitude of house-keeping genes. However, Phylo SI was also able to identify organisms with exceptional genome architecture that were not singled out by the standard approaches. In the second data set, Phylo SI resolved several uncertain relationships, such as in the *Brucella* clade, which could not be identified by the other approaches due to their weaker phylogenetic signal.

The method with an accompanying documentation and the data used for this study is available at http://research.haifa.ac.il/ssagi/software/PhyloSI.zip. Supplementary material used in this study is available at http://research.haifa.ac.il/ssagi/SI/sup.zip

## MATERIALS AND METHODS

### Preliminaries

**Figure 1.**
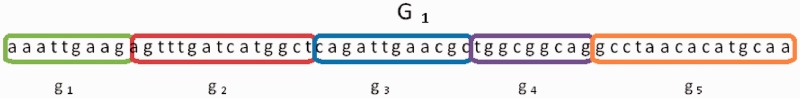
A genome is viewed as a sequence of genes, while a gene is a sequence of nucleotides.

**Figure 2.**
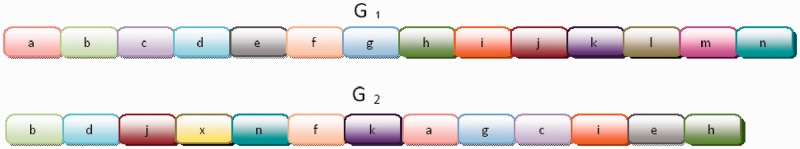
Comparing *G*_1_ with *G*_2_ for *k* = 3: 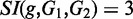, 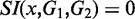, 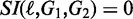.

A genome is a sequence of genes 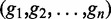 and each gene is a sequence of DNA letters. That is, our view of a genome is at a resolution of genes, and of a gene at a resolution of nucleotides (see Figure 1). The ‘*k*-neighborhood’ of a gene *g*_0_ in genome *G*, 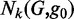 is the set of genes at distance at most *k* from *g*_0_ in *G* (i.e. at most *k* genes upstream or downstream). The conservation of gene order between two genomes is called ‘synteny’. Let *g*_0_ be a gene common to two genomes 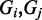. Then the ‘*k* synteny index’ (*k*-SI), or just SI when it is clear from the context, of *g*_0_ in 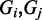 is the number of common genes in the *k*-neighborhoods of *g*_0_ in both *G_i_* and *G_j_*: 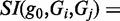 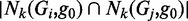. We note that in cases of circular genomes, a genome is broken arbitrarily at some location and the *k*-neighborhood should be taken accordingly (i.e. circularly). For the sake of completeness, for 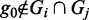, 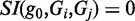. See Figure 2 for illustration.

**Figure 3.**
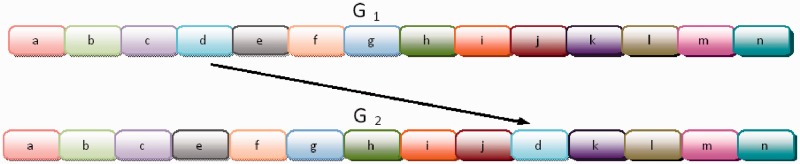
Gene *d* was transferred from Donor species *G*_1_ to recipient species *G*_2_.

A genome undergoes events of gene gain and loss in which genes are added or removed respectively. These events produce variation over the gene repertoire of the various genomes. A HGT is defined as an event in which a gene of a genome, the ‘donor genome’, is copied and inserted at some position in another genome, the ‘recipient genome’. Because we view the genome as a sequence of genes (see Figure 3), the new gene is always between two genes (or at the ends of the genome).

### SI-based phylogenies

We start this part with an overview of the method proposed. In the event of HGT, a gene is being inserted at the recipient genome. That gene either did not exist in the recipient genome or has functionally replaced the old copy (otherwise it is not considered HGT by our definition). The probability that the gene maintains in the recipient genome its old *k*-neighborhood, or even part of it, in the donor is at the order of 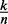. As we choose 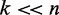, this probability is small. The above can be extended to the case of HGT of operons or gene clusters, in which a sequence of neighboring genes with a similar or related function, located next to each other in the genome, are being copied. Therefore, SI of a specific gene gives a measure of the likelihood of that gene being horizontally transferred. Nevertheless, synteny between genomes decreases with time as a result of large-scale mutational events. Thus, when the whole genome has low SI, we cannot take low SI of a certain gene as indicative for HGT. However, we can use the SI to measure distances between the genomes exposed to high HGT activity. We seek a measure that will consider the SI of all genes in the genome.

**Definition 1**

Given two genomes 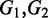, and let 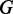 be the set of genes in at least one genome, 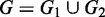. Then the average k-SI between *G*_1_ and *G*_2_ is defined by
(1)
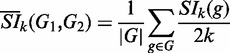

We observe that for two identical genomes, 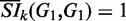, and for two genomes with disjoint sets of genes, 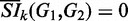. The ‘SI’ therefore gives us a measure of similarity between pairs of species, which we can use to construct evolutionary trees over the whole set of species. This property is attractive in particular for the organisms we investigate, as they are subjected to heavy HGT activity resulting in different histories for different sets of genes. Therefore, a method considering the aggregate set of genes is required. It is important to note here that the SI for a gene is not binary, i.e. either 1 or 0, as a result of being transferred or not. Genes can have SI values that range anywhere between 1 and 0 as a result of either being transferred with part of their original neighborhood, or they could have kept their original neighborhood, but that neighborhood was additionally affected by other HGT events.

When applying 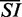 between all species, we obtain a similarity measure between the set of species. We can use this measure for phylogenetic reconstruction if we convert it to distances between the species. Hence we define 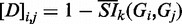 as our distance metric. Note that every entry in *D* is between zero and one. Once we have the distance matrix *D*, we can use it to construct a phylogeny from it. Distance-based phylogenetic reconstruction methods receive as input a symmetric dissimilarity matrix, representing dissimilarities between the taxa set under study, and strive to return a tree over the taxa set, such that tree distances (i.e. the path lengths) between the leaves, best approximate the distances in the matrix (see more details in the Supplementary Text). For our task, we used the neighbor-joining (NJ) (45) algorithm implemented in the Phylip package (46).

### Bootstrap

To perform bootstrap analysis, we devised the following approach to allow bootstrap for the new method: for every pair of genomes 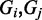 from the genome set 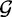, we constructed the SI distribution *f*(*SI*), where, for 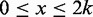, *f*(*x*) holds the number of genes in the union set of genes 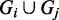 with *SI* = *x*. Next we conducted a weighted sampling from that distribution with number of samples 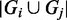. Having done so for all pairs, we obtain the ‘bootstrap SI matrix’ from which we built the tree. A more detailed description is found in the Supplementary Text.

### Simulation procedure

We used simulations to compare the new SI based-method to other comparable methods operating on genomic data. Here we briefly describe the simulation procedure. A fuller description of the algorithm can be found in the Supplementary Text. Our basic assumption is that the gene gain and loss are time-dependent Markovian events with some constant rate, operating on the species (47). Therefore, we generated a random ultrametric species tree by the Yule process (48) describing the speciation history of the species set. Edge lengths in this tree represent the expected time until an event under a Poisson process and hence were distributed exponentially with parameter 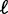. Based on this species tree, we simulated a Poisson process of gene gain/loss with a constant rate of events. We start with an ancestral genome as a string of genes at the root of the species tree. Events were generated on an edge with probability proportional to the time between speciation (edge length). Once an event is generated, with probability *pHGT* it is a gene gain and with probability 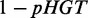 it is a loss.

As gene gain may sometimes result in a replacement rather than a real gain, we tried to calibrate the parameters such that our genomes do not shrink too much at the leaves.

Therefore, the input to the competing methods included the resulting genomes at the leaves, and the trees reconstructed by the various methods were compared with the model species tree.

### Tree similarity measures

There are several approaches to measure similarity between phylogenies. These are normally used in simulation studies where the ‘true’ model tree is known and the accuracy of the reconstruction method is measured by the distance of the reconstructed tree to the model tree. There are several tree metrics. We chose the most common ones: (1) ‘Robinson-Foulds (RF) Symmetric Difference’ (49) counts the number of different edges between two trees implemented in Phylip (46). We used a variant measuring similarity instead of difference. (2) ‘Maximum Agreement Subtree’ (MAST): The largest subset of the taxa set, under which both trees are the same. (3) ‘Quartet Fit’: The number of identical induced quartet trees (out of the total number of induced quartet trees). Full detail on the above measures appears in the supplement.

### Reconstruction methods

The following reconstruction methods we compared in this study. We used the Phylo SI method described above with *k* = 10 based on the simulation results and biological rational. The GRAPPA toolbox for inversion/breakpoints distance-based phylogenetic reconstruction was taken from (24).

We also implemented two additional whole-genome–based methods to compare with the former two:

- Directed Pairs (DP): As suggested in (33), we counted in two given genomes the number of gene pairs that exist in both genomes and the open reading frames they encode are in the same orientation.
- GC: This approach sets the distance between two genomes as the fraction of genes residing in only one genome divided by the size of the union set of genes in the genomes. We note that this approach is a special case of the SI-based method with 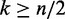.

### Data sources

All genomes analyzed were downloaded from the NCBI microbial genomes resources (50) (http://www.ncbi.nlm.nih.gov/genomes/lproks.cgi). A set of 89 bacterial organisms representing the major clades of the bacteria domain with completely sequenced and annotated genomes was randomly selected.

The Tree of Life (TOL) topology for these organisms was extracted using the iTOL tool (51).

Appropriate 16S-rRNA genes were downloaded from the Ribosomal Database Project (RDP) (52–54). RDP provided two sources for trees, namely a distance-based ready-made tree for selected organism and prealigned sequences, based on rRNA secondary structure alignment, that are available from RDP for further independent comparative analysis (including phylogenetics). As the maximum likelihood (ML) reconstruction is considered more reliable than distance based, we chose to use the aligned sequences and in the Supplementary Material we provide also results from the NJ tree from RDP. We applied a ML reconstruction under the GTR + Gamma evolutionary model (designed for sequences with significant between-site rate heterogeneity), using PhyML (55) on the aligned sequences. A variant of the 16S-rRNA tree is the AMPHORA tree that instead of relying on the16S RNA gene alone, uses a multitude (31) of highly conserved proteins, with manually curated alignments (2). We extracted the AMPHORA tree over (or induced by) our taxa set from (56).

Finally, we constructed three whole-genome–based trees using the methods outlined above: SI, GC and DP. The names and order of genes were extracted using RefSeq annotation (57), as it provides an easy to use source of such data. We are aware that RefSeq is not adequate for this task and for a more comprehensive study, a better database, constructed using a more appropriate orthology detection tool should be used. We applied a preprocessing stage to the gene lists extracted from RefSeq in which spurious genes were removed. Full details and statistics about this stage appear in the Supplementary Text. Additionally, to account for possible inaccuracies in RefSeq, we set the value of k to 10. The main weakness of RefSeq in the context of this study is its partial coverage for some genomes. However, by excluding these genomes from the analysis and associating confidence to each node, this problem is alleviated.

## RESULTS AND DISCUSSION

Validation of a new phylogenetic method requires its comparison with widely accepted ways of tree reconstruction. We implemented our method in software and tested it in various simulations and real data environments.

### Simulation study

#### Choosing the Optimal k

Our first attempt was to study the behavior of the SI measure as a function of evolutionary distance. Our basic assumption is that the probability of a HGT at a gene grows as a function of time (34), and therefore, a gene at two genomes will have a greater probability to reside at different *k*-neighborhood, the longer the time between divergence of the two genomes. Hence, we attempted to quantify how this probability affects the separability of two genomes as a function of *k*. In other words, we sought to determine the values of *k* under which we will have the most distinction between genomes separated by different times.

**Figure 4.**
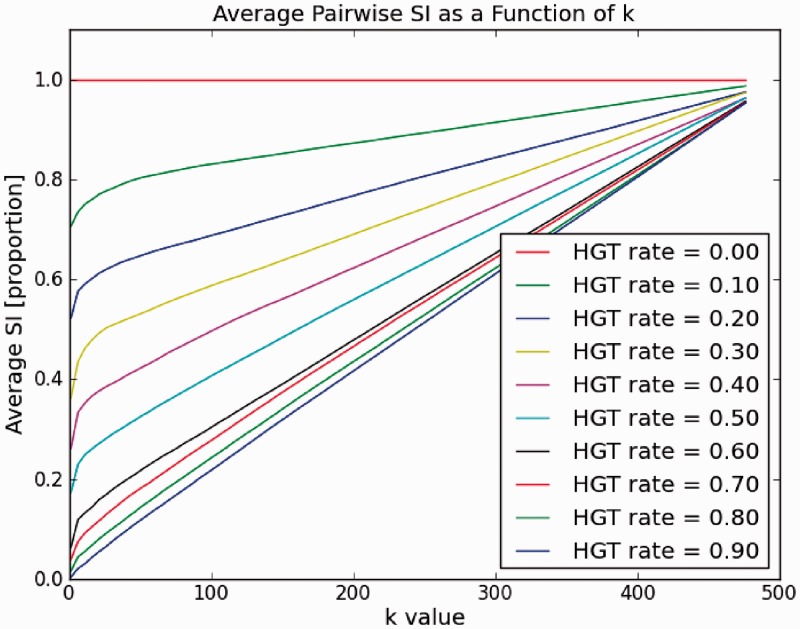
SI between a pair of genomes (#genes per genome = 500). Measured SI as a function of *k* for various HGT rates (HGT probability at each a gene in a genome).

To answer this question, we conducted the following experiment. We applied a constant ‘HGT rate’ to a genome, that is, we perform a HGT event at a gene in a genome with a constant probability *P* for every gene in the genome, where *P* represents the HGT rate. Hence, the rate of HGTs at a gene in that genome is *P* (this should not be confounded with the rate describing edge lengths as described below). We wanted to measure how the SI changes as a function of *k*. We repeated this procedure for several values of *P* representing genomes at different evolutionary distances. The results are shown in Figure 4.

As can be seen from the figure, the SI is small for closely related genomes and larger for more distant ones. Hence, we can infer that the SI measure is a good indication for evolutionary distance, and according to our model, it can serve for phylogenetic reconstruction. Next we sought to identify the optimal *k* providing the best separability for all distances. We see that at higher values of *k*, SI is similar for different values of *P* and approaching 1 when *k* approaches 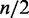. It can also be noted that, as the curves are not linear, greater separability is achieved at lower *k*-values around 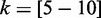. Thus, given a set of genomes, the optimal *k* should be determined as value that maximizes simultaneously the separability between all pairs, and at the same time is not too small to account for artifacts caused by incorrect orthology detection or other factors. This can be found by simply applying the method, without reconstructing a tree, for several values of *k*.

#### Using the SI measure for whole-genome–based phylogenetic reconstruction

Based on the previous insight, we can use the SI measure to reconstruct trees. Hence, we define the synteny distance as 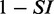 and used this dissimilarity measure under a distance-based method. Specifically, we used the NJ algorithm (45) for this purpose as described under ‘Materials and Methods’ section. Our goal in this part is to gauge the sensitivity of the tree reconstruction precision to different values of *k* and to the relative mutability of the gene.

**Figure 5.**
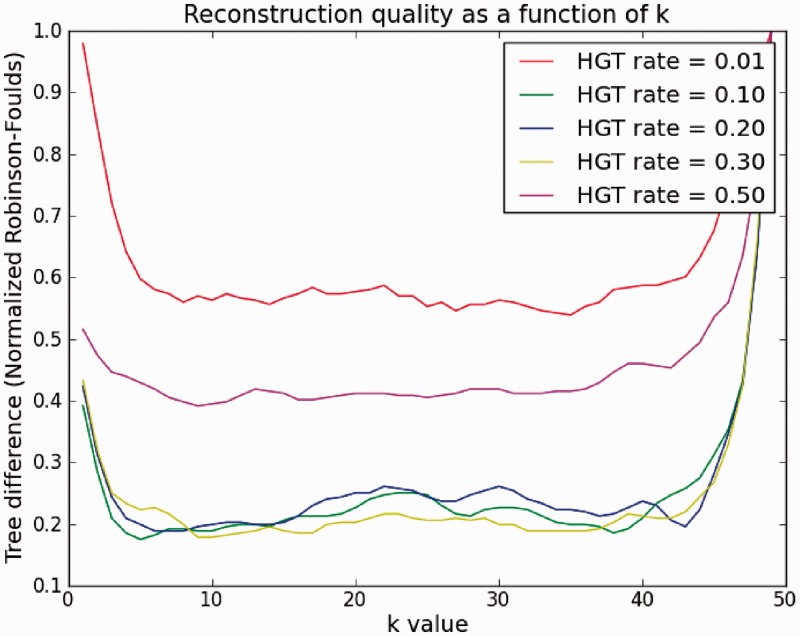
Quality of reconstruction (RF symmetric difference to the model tree) as a function of *k* for various HGT rates (HGT probability at each gene in a genome). Simulated number of taxa (n) is 100, genome size is 500.

Hence, we simulated trees with different average mutation rate per edge (See ‘Materials and Methods’ section) and applied the SI method for tree reconstruction. In Figure 5, we kept the average rate of HGT (HGTs per a gene in the genome) constant. This is the parameter for the exponentially distributed tree edge lengths (see Supplementary Material for fuller details). Thus, at each edge in the tree, the probability of a gene to undergo HGT, i.e. to be acquired horizontally, is distributed exponentially with the HGT rate as a parameter (this can be denoted as the inverse of ‘edge length’, but for simplicity we defer it for a later stage). We applied the SI algorithm each time with a different *k* on the resulting genomes at the leaves of the tree. The curves show the percentage tree difference (RF, see ‘Materials and Methods’ section). We see that under all HGT rates, a value of *k* = 10 is sufficient for accurate reconstruction. Another interesting, albeit expected, result is that for very high or low rates of HGT, only poor reconstruction is obtained (∼0.4 or 0.6%, respectively) regardless of the value of *k*. This is explained by the fact that for a very low HGT rate (the curve corresponding to HGT rate 0.01), there is no strong enough signal to distinguish between the leaves (genomes), in particular close ones. On the other hand, when the rate is too high (curve corresponding to HGT rate 0.5), genomes are saturated with HGT and the power of the method decreases. However, as our results on real data show, such high HGT rates are rare, so we are in the ‘safe zone’.

In the Supplementary Text we also show a similar result from a different perspective, for better illustration.

#### Comparison to genome rearrangement software

The genome rearrangement problem is defined as finding the shortest sequence of rearrangement operations for converting one genome to another. Similarly to the SI, this measure (the number of operations) can be viewed as a distance between genomes and hence be used for phylogenetics (27). Although the two measures, the SI and the rearrangement distance, measure different processes, some special cases of both problems exist and we can confine a comparison of the two methods to these cases.

**Figure 6.**
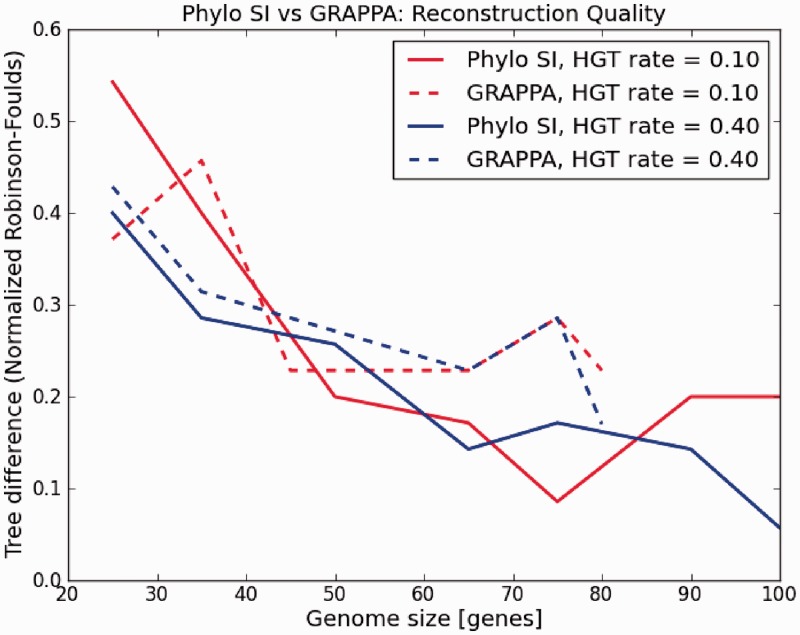
‘Phylo SI’ versus GRAPPA reconstruction difference for simulated phylogenetic trees. Genome sizes = [25, 100], HGT rate = [0.1, 0.4], number of taxa in the phylogenetic tree = 10, *k* in ‘Phylo SI’ = 10.

**Figure 7.**
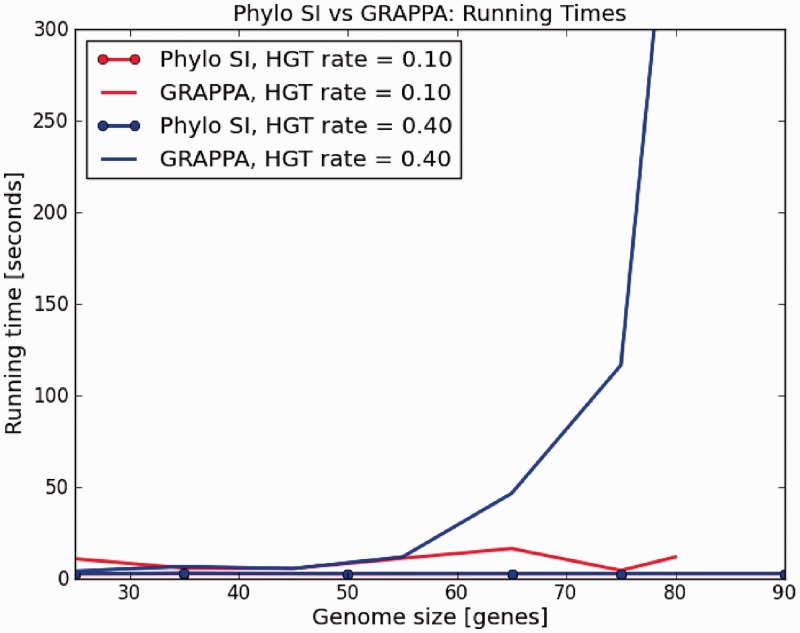
‘Phylo SI’ versus GRAPPA running times. Genome sizes = [25, 100], HGT rate = [0.1, 0.4], number of taxa in the phylogenetic tree = 10, *k* in ‘Phylo SI’ = 10.

The main disadvantage in the genome rearrangement problem is the restriction that the two compared genomes must have the same set of genes [but see (28) for extensions of this approach]. Hence, under this model, a HGT can be perceived as a translocation in one genome. To compare Phylo SI to such a technique, we chose the software GRAPPA (24), one of the most popular implementations of genome rearrangement algorithms. As GRAPPA is slow, the study was restricted to unrealistic tree sizes of 10 species and tiny genomes of up to 80 genes. Accuracy (RF distance) was measured as a function of genome size. The results are shown in Figure 6. Running times were also compared as shown in Figure 7. From the figures we can see that Phylo SI is at least as good (accurate) as GRAPPA, regardless of the rate of HGT, and the advantage grows with the size of the genome. Moreover, running times of GRAPPA became prohibitive for genome sizes >80 genes. We also see (Figure 7) that running times of GRAPPA grow exponentially with both the size of the genome and the rate of HGT. This limitation makes GRAPPA useless for large-scale analyses of hundreds of species.

### Simulation results with related whole-genome–based reconstruction techniques

**Figure 8.**
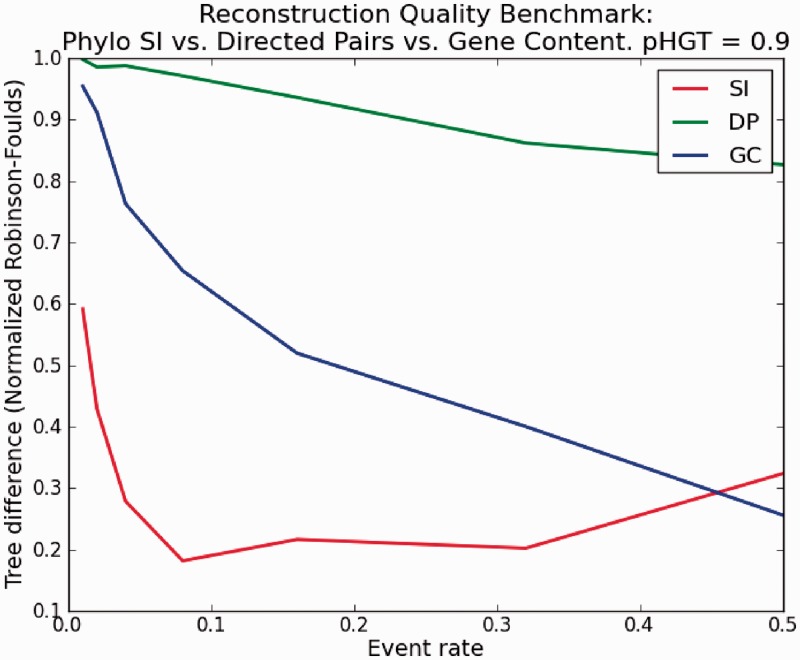
Phylo SI versus. DP versus GC reconstruction quality Benchmark.

Two other whole-genome–based approaches that were suggested in the past are DP (33,58) and GC (29). In the DP approach, the number of ordered uninterrupted gene pairs that are present in both genomes is divided by the total number of pairs shared by the two genomes. This measure reflects the degree of gene order similarity between the genomes, and as with the SI, we subtract it from one to convert it to a metric. The other approach is the traditional GC that simply counts the number of shared genes, normalized by the size of the union gene set in the two genomes. As these methods are affected by both gene order (DP) and gene gain/loss (GC), our simulation procedure combined the two processes, namely gene gain/loss and HGT (see ‘Materials and Methods’ section). We aimed at simulating as similar as possible a process to the real bacterial genome data we analyzed (minimal size of gene intersection set between two genomes 0.18 of genome size, average size of gene intersection set between organism 0.35 of genome size, see section on real data analysis) so we set our parameters accordingly. Figure 8 shows the results of this analysis. In the supplement we provide fuller details on the parameters used and show similar results for more values of pHGT. We set the ratio of HGT to gene loss as a constant and varied the event probability at an edge. It can be seen that the DP approach is significantly inferior to SI and GC, whereas for the relevant values of parameters, SI is superior to GC. We note that for high rate of events at an edge, the GC approach outperforms SI; however, at such rates, the resulting genomes at the leaves are small as a result of heavy gene loss events (only a few genes) and the signal is weak.

### Real data analysis

We also applied our method to real genomic data. Here we separately analyzed two types of data: a large, diverse, arbitrarily selected bacterial data set and a smaller data set of Alphaproteobacteria for which there are published results regarding its evolutionary history. As here we cannot compare the result of Phylo SI to a model tree from which the data were generated, for both data sets we contrasted the SI tree with other published trees over the same taxa set. We made several comparisons and further analyses on each tree separately and also between the trees. We note that the comparison of the SI tree to the other methods, as opposed to the simulation study, should not indicate the accuracy of the method, as these methods measure different processes and hence result in different trees. Moreover, a dislocation of a specific taxon can point out to some irregularity in its genome architecture, as we next show. Therefore, the part of study provided here allows for a noncommon comparison between several trees (i.e. evolutionary hypotheses) for this set of organisms.

All trees in Newick format appear in the Supplementary Material.

### Large, diverged, uniformly selected bacterial set

**Figure 9.**
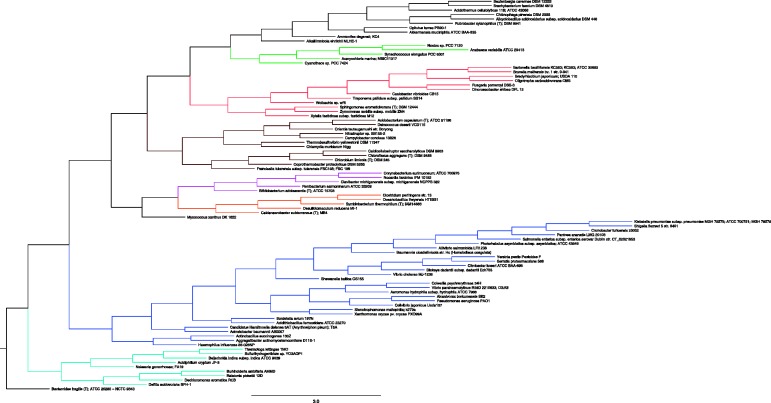
The SI tree on 89 microbial organisms. The tree was constructed by NJ from pairwise distances 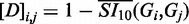. The tree is, by construction, fully resolved (86 internal branches).

The data set to which we applied our method is a set of 89 fully sequenced bacterial species chosen uniformly and arbitrarily from the NCBI genomic database (59). For each pair of genomes 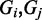 we constructed the average synteny - 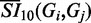 (i.e. the 10-neighborhood of every gene is taken) and generated the induced dissimilarity matrix. Finally, we used the standard NJ algorithm (45) implemented in Phylip (46) for constructing phylogenies from dissimilarity metrics. The resulting tree is shown in Figure 9 with major phyla color-coded.

Although the tree contains several branches that correspond to known phyla [e.g. Firmiicutes (orange), Actinobacteria (pink) and Cyanobacteria (green)] and correctly places many sister taxa, it nevertheless has several inconsistencies with known taxonomic relations of bacteria. One large clade (marked in blue), divided into two sub-clades contains only gammaproteobacteria, but some gammaproteobacteria branch deeper with *Bordetella*, which is a beta-proteobacterium. Failure to separate these closely related classes is common in both genome-based analyses (36) and rRNA-based analysis (60). However, there are also more extreme cases such as members of the Aquificae and Thermotogae phyla that fall within the betaproteobacteria branch (cyan). As can be also seen from the figure, there is an uncolored clade (on the top) containing taxa from unrelated groups. A closer examination of the genomes in that clade reveals that few of them contain only a handful of annotated genes under RefSeq, with 14 genes for the *Alicyclobacillus acidocaldarius* as an extreme case. Needless to say, any reliable inference is impossible in such cases. Whereas we address this problem separately at a later stage, we now compare this tree with other trees over this taxa set notwithstanding the above exceptions. We contrasted the resulting tree with three other ‘accepted’ trees over this set of species: the TOL extracted from the iTOL (1), a 16S-rRNA–based tree constructed using ML approach from aligned sequences extracted from the RDP database (52), and finally, the tree constructed with AMPHORA suit (56). The three additional trees, the TOL-tree, the 16S-rRNA tree and the AMPHORA tree, are shown in Supplementary Figures S10–S12, respectively, in the Supplementary Text. Each of these other trees have some limitations. The TOL was constructed from an aggregate of genes from which putative cases of HGT were removed (1). The result is a partially resolved tree with only 41 internal branches (splits) out of the maximum possible 86. In contrast, the 16S-rRNA tree is fully resolved (a binary tree with 86 internal edges); however, even a highly conserved gene such as the 16S-rRNA was found to exhibit confounding evolutionary histories for certain bacterial organisms [e.g. Cyanobacteria (8,61,62)]. Moreover, due to its conservation, the 16S-rRNA might not convey enough information to distinguish between close species or even strains (as we show below). Finally, although the AMPHORA tree was made with the purpose of alleviating the 16S-rRNA tree drawbacks by concatenating several protein coding genes, it is not immune to errors such as HGT, sequence alignment artifacts, and so on. Similarly to the TOL, AMPHORA also relies on highly conserved genes and, moreover, on their protein sequences, and hence may not convey enough signal to distinguish between closely related species or strains.

We applied the three tree similarity measures discussed in the ‘Materials and Methods’ section between all pairs of the above four trees. The results are depicted in Table 1. We note though that one should take these comparisons with caution, as they measure different evolutionary mechanisms that may differ greatly within wide range of species, and hence cannot indicate on some ‘correctness’.

**Table 1.** Pairwise similarities between the four trees

	Tree Similarities			
	16S	AMPHORA	ToL	SI
16S	100/100/100	53/91/67	12/51/42	15/69/49
AMPHORA	53/91/67	100/100/100	13/51/41	14/66/52
ToL	12/51/42	13/51/41	100/100/100	14/61/62
SI	15/69/49	14/66/52	14/61/62	100/100/100

Each entry contains the percentage similarity according to common edges (RF), quartet fit and MAST, respectively.

The first measure is the percentage of common edges between the trees (denoted by RF, as it is defined by one minus the RF distance). As we see from the table, there is a higher similarity between the 16S-rRNA and AMPHORA trees. However, although the other RF scores appear to be fairly low, we now show they are far from being incidental. To estimate the significance of these low RF scores, we need to compare it with a null model—a random tree. In (63) asymptotic results for the distribution of distances between random trees are studied. We, however, deal with a specific tree, and of relatively small size. Hence, to test the significance of this result we pursued the same approach as (64). We generated 100 000 pairs of (binary) random trees of the same size and calculated the distribution of the RF over these random trees (see histogram in Supplementary Material). In 80% of the cases, the trees had not any edge in common and 7% edges in common only 0.002%. Needless to say that a similarity of 12% common edges between the random trees was never encountered.

The RF distance is a strict measure in the sense that a small perturbation in the tree, e.g. a relocation of a single species in the tree, decreases the score substantially. It was also argued (65) that RF distances favor methods that produce unresolved trees, and hence can tend to make methods that return consensus trees look more accurate than methods that produce binary trees. This is in particular relevant due to the fact that the TOL tree is so loosely resolved.

A more tolerant measure is the ‘quartet fit’ (66) in which the topologies of all 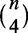 possible quartets are compared between the trees. A quartet agrees with a tree if the tree induces the same topology over the same four taxa as in the quartet (see definition above). As 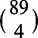 is too large a number to be analyzed, we generated a random sample of 1 000 000 quartets from one tree and compared it with the other. The results appear at the second row of Table 1. As can be seen, the SI tree resembles each of the other trees more than they resemble each other. To assess the significance of these results, we calculate the probability of such scores to be obtained by chance. For this purpose we first define a ‘random tree’:

**Definition 2**

A binary unrooted random tree *T^r^* over *n* species is obtained recursively from a random tree over *n* – 1 species by choosing uniformly at random, any of the 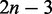 edges, splitting it by a new internal node and attaching (by an external edge) the *n*th taxon to that node.

The process is started from an unrooted tree over three taxa, which is a star tree with one internal node and three external edges. It can be easily shown that any binary tree on *n* nodes is obtainable by this process, and all trees are equiprobable. Moreover, a quartet tree (e.g. 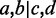) is consistent with a random tree with probability 1/3 (as there are three topologies and they appear in the same frequency in all trees). Now, let *Q*(*T*) be the full set of 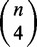 quartets induced by a binary, unrooted tree over *n* taxa. Then, the above implies that the expected number of quartets from *Q*(*T*) satisfied by a random tree is 
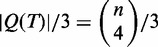
. Hence, the quartet fit significance score is defined as follows:

**Definition 3**

(quartet fit significance) The significance of quartet fit score *s* is the probability to obtain a (quartet fit) score at least *s* on a random tree *T^r^* (for a given quartet set *Q*).

We note that the topology of *T* is not important, as *T^r^* is random and hence, the quartet fit significance is the same for any tree *T*. To answer this question, we use bounds on large deviations for the binomial distribution (67) as follows. Let 
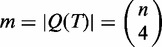
 and *S_m_* is the number of quartets satisfied by *T^r^*. Now, by the definition of *T^r^*, every quartet is considered as a Bernoulli trial with success probability 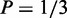. The probability of obtaining a success ratio of at least *a* in a series of *m* trials is bounded by
(2)
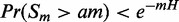

where 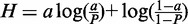 is the relative entropy between a p-coin and an a-coin (also known as the Kullback–Liebler distance). As this bound diminishes exponentially in the number of trials *m*, it is easy to see that a success ratio of 60% versus random 1/3 in 1 000 000 trials is undoubtedly significant.

Finally we applied the MAST tree metric as described in the ‘Materials and Methods’ section. We conducted the same simulation as in the RF to assess the significance of these results. The complete histogram appears in the Supplementary Material. It can be evidenced that the MAST results show fairly high similarity between the trees, significantly above random similarity, where the value of 0.4 was hardly achieved by random.

To further validate these results we also compared three more trees: The original 16S-rRNA tree from RDP, built by NJ, and the DP and GC trees from the RefSeq data used by the Phylo SI method. The three complete seven by seven matrices, corresponding to each of the three tree metrics, appear in the Supplementary Material. These additional data suggest that there is a relative higher similarity among the trees based on sequence (DNA, proteins) data (the RDP and AMPHORA trees) and also among whole-genome–based trees (SI, DP and GC trees) and lesser between members of different groups (although the SI tree also exhibits a high similarity to these ‘sequence-based’ trees). The TOL tree in general exhibits lower similarity to all other trees.

As indicated above, the taxa set used in this part contained several species with poor RefSeq annotation. This not only produced more or less random results but also cannot be reflected in the bootstrap approach developed for the method, rendering these values futile. To cope with the latter problem, we pursued the following approach. We filtered out all genomes with <500 genes and applied Phylo SI only to the remaining genomes. We also developed a recursive ‘confidence value’ for a node in the tree, based on weighted average, aimed at replacing the bootstrap value for this setting. This confidence value reflects the ‘coverage’ of the annotation at the genomes, in terms of the fraction of annotated genes from the total number. The tree along with the confidence values appears in the Supplementary Text in Supplementary Figure S13, as well as details about this new criterion.

The SI tree is generally in good agreement with expected phylogenetic relationships, with the phyla Actinobacteria, Cyanobacteria both forming monophyletic clades, and the classes Gammaproteobacteria and Alphaproteobacteria being monophyletic, with the exception of *Francisella tularensis* and *Orientia tsutsugamushi*, respectively. These two exceptional taxa have genomes riddled with repetitive elements: *O. tsutsugamushi* has ∼4200 identical repeats of >200 bp in size, which account for >37% of its genome (68), whereas *F. tularensis* has 50 copies of the transposon ISFtu1 and a duplicated region of 33.9 kb (69). The presence of a large number of repetitive elements can generate homologous recombination events and randomize gene order within the genome, especially in intracellular pathogens, such as *F. tularensis* and *O. tsutsugamushi* that tend to have relaxed selection pressures on genomic changes and mutations alike. This randomization of gene order probably obscures the synteny-based phylogenetic signal in these species. On the one hand, this is a limitation of the Phylo SI method but on the other hand this makes the method particularly useful for identifying such unusual genomes. Importantly, phylogeny within the gammaproteobacteria is well resolved.

Summarizing the results for this part, it is evident that there is some incongruence between the trees constructed by these three different approaches that do not necessarily reflect superiority of one approach over another, rather due to differences among the methods that are sensitive to various types of ‘noise’. Further, the similarity between trees built by the same approach (either nucleotide based, or gene based), but not between those from different approaches, emphasizes the existence of different processes in the evolution of genome architecture (70,71). This may suggest a more inclusive attitude toward phylogenetics in prokaryotes than reliance on sheer sequence similarity between homologous genes.

### Alphaproteobacteria

Our second benchmark for the SI-based method was to test it on a less-diverged bacterial class that have been well studied in physiologic and taxonomic contexts. This analysis facilitates comparisons between methods in a biological context, including those where the best known phylogenetic marker, the 16S-rRNA, does not provide sufficient taxonomic resolution.

We therefore chose a set of 45 Alphaproteobacterial species. As before, we also constructed the TOL tree by means of iTOL (51) and the 16S-rRNA gene-based tree (see ‘Materials and Methods’ section) using RDP.

The three trees—the SI-tree, the 16S-rRNA tree and the TOL tree, are depicted in Supplementary Figures S14–S16, respectively, in the Supplementary Text. Both the 16S-rRNA–based and the TOL trees contain unresolved branches, especially more recently diverged clades within the trees (e.g. the genus *Brucella*). These may be the result of confounding evolutionary histories for different genes (TOL) due to HGT or to the lack of evolutionary signal due to insufficient number of substitutions (16S-rRNA and TOL).

As we are interested in the biological relevance of the resulting trees, we set to perform bootstrap analysis to establish branch support in the trees. The TOL comes without bootstrap values and the sequences are unavailable and therefore was excluded from this further analysis. Hence, we restricted the bootstrap analysis only to the 16S-rRNA and SI trees. The 16S-rRNA bootstrap was constructed with the correction for multiple substitutions and excluding positions with gaps, whereas the SI bootstrap was constructed as described above (see ‘Materials and Methods’ section). For both trees, branches with <80% bootstrap support values were collapsed, as shown in Supplementary Figure S17 at the Supplementary Text. In terms of branch support, the SI tree was superior to the 16S-rRNA tree by preserving 32 versus 26 branches (20% more). Although it is a common practice to manually remove ambiguous positions from multiple sequence alignments, resulting in much improved bootstrap values, this requires significant know-how and skill. Thus, having a fully automated method, such as Phylo SI, capable of generating highly resolved phylogenies without user intervention, can be a significant improvement, especially in a clinical setting.

Generally, the deep relationships, such as those between families and orders were well recovered in all trees (e.g. Rickettsiales, Rhizobiaceae or Brucellaceae), and thus all methods were fairly accurate. Nevertheless, some mismatches were found. For example, *Agrobacterium radiobacter* K84 was sister taxon to several *Rhizobium* species in the SI and 16S-rRNA–based trees, but not in the TOL, where it clustered with other *Agrobacterium* species. This inconsistency may be explained by a recent study (72), which suggests that *A. radiobacter* K84 should actually be reclassified as *Rhizobium rhizogenes* K84 and does not actually belong to the genus *Agrobacterium*.

On the other hand, the clade containing the genus *Brucella* is well-resolved by the SI tree but not in the 16S-rRNA based or TOL trees. This classification was compared with an established tree, based on sequences of multiple manually selected genes (73) and the two have the same topology for the *Brucella* species examined. Moreover, the TOL tree did not reconstruct an informative subtree for the *Bartonellaceae* family, whereas both the 16S-rRNA–based and SI trees successfully resolved it, obtaining concordant topologies.

In addition, the Pathosystems Resource Integration Center (PATRIC) (74) provides analysis tools and a rich database for all bacterial species in the selected the National Institute of Allergy and Infectious Diseases categories A–C priority pathogens list. This database has an implemented phylogenetic pipeline that reconstructs organismal phylogenies based on a concatenation of reliable residues from many proteins shared by the taxa in question, not only the highly conserved ones that are included in ITOL. PATRIC was used to construct a phylogenetic tree containing 24 out of 45 Alphaproteobacteria examined above. A comparison between the SI and the PATRIC trees reveals two disagreements, namely the positions of *A*. *radiobacter* K84 and *Sinorhizobium meliloti* 1021, where PATRIC supports the iTOL topology (see above) over the SI’s topology. However, the *Brucella* clade in both trees had the same topology, apparently demonstrating the correct solution obtained by the SI method, in a case where iTOL and 16S-rRNA–based trees fail.

In summary, our findings indicate that Phylo SI is particularly useful in resolving the accurate phylogenetic location of species within genera. This circumvents the weak signal provided by many slow-evolving genes commonly used in phylogenetic analyses. The method can also suggest alternative explanations to traditional beliefs of species evolution that are based on information orthogonal to sequence based similarities.

## CONCLUDING REMARKS

In this work we have described a new approach for phylogenetic reconstruction, Phylo SI, that appears to be useful, in particular, for groups of organisms characterized by high gene mobility. The method is based on the conservation of gene order among species but, as opposed to other existing gene order–based methods, takes into account also events of gene gain/loss. Phylo SI defines the SI that captures the relative synteny conservation between two organisms and averages this value across the whole genome. The Phylo SI method provides a quick and efficient way to reconstruct the phylogeny of a large number of organisms for which genome sequencing data are available. With rapidly decreasing sequencing costs, bacterial genome sequencing in emerging pathogenic isolates is becoming routine, further emphasizing the need for quick and accurate taxonomic placement of the bacteria in question. The SI method only requires the locations of genes in the genome and can provide independent validation of the commonly used sequence-alignment–based phylogenies, such as those based on rRNA gene alignments or concatenation of multiple genes (2), or the iTOL (51).

The Phylo SI method is parameter and model free, and does not require any previous knowledge, manual selection of genes, multiple sequence alignments and their refinement or lengthy computation. Moreover, it is well grounded in evolutionary principles. Far from being random, the order of genes in the genome, known as the genome architecture, is substantially conserved in microbial evolution, probably reflecting selective pressures (75).

We show by simulation the advantages in resolution over less sensitive approaches such as gene presence/absence (29) or DP (33) and in performance over genome rearrangement software. Our real data results suggest the existence of a distinct process of genome architecture evolution that does not necessarily conform with the evolution of single genes, even the more conserved ones. We also demonstrated the power of this method in resolving the taxonomic placement of species within genera, which is highly useful for bacterial taxonomy, and can be applied rapidly and without prior knowledge, which may be important in a clinical setting. Furthermore, because this method can provide additional independent support for clades where existing methods disagree (see above), it represents a useful addition to the current phylogenetic toolkit available to microbiologists.

## SUPPLEMENTARY DATA

Supplementary Data are available at NAR Online.

## FUNDING

Funded by Israel Science Foundation, University of haifa, and the Israel/USA Binational Science Foundation (in partial) (to A.S., N.N. and S.S.). Funding for open access charge: Israel Science Foundation, University of haifa and the Israel/USA Binational Science Foundation.

*Conflict of interest statement*. None declared.

## Supplementary Material

Supplementary Data
