# Supplementary material for: Phylo SI: a new genome-wide approach for prokaryotic phylogeny
Source: Nucleic Acids Res. 2013 Nov 15;42(4):2391–404. doi: 10.1093/nar/gkt1138 (PMC3936750; doi:10.1093/nar/gkt1138)
Supplement: Supplementary Data [file supp_42_4_2391__index.html]

Phylo SI: a new genome-wide approach for prokaryotic phylogeny — Phylo SI: a new genome-wide approach for prokaryotic phylogeny — Supplementary Data 

# Phylo SI: a new genome-wide approach for prokaryotic phylogeny

## Supplementary Data

files

**Files in this Data Supplement:**

- Supplementary Data - pdf file
