# Supplementary material for: Phylo SI: a new genome-wide approach for prokaryotic phylogeny
Source: Nucleic Acids Res. 2013 Nov 15;42(4):2391–404. doi: 10.1093/nar/gkt1138 (PMC3936750; doi:10.1093/nar/gkt1138)
Supplement: Supplementary Data [file supp_gkt1138_nar-02397-n-2013-File002.pdf]

supplementary Material

October 14, 2013

1 Phylogenetic Reconstruction

Since the second stage in our algorithm builds a tree from a distance matrix, and since we also compare our trees to competitive trees constructed by standard approaches, we here give a very high level description of phylogenetics. Standard sequence (or nucleotide) based phylogenetic reconstruction receives as input a set of n orthologous sequences (typically representing the same gene at the n different species under study) and attempts to constructs a tree that best represents the evolutionary history of the gene set. These methods are divided into two main categories: character based versus distance based methods. Character based methods first align the sequences by stretching them to a uniform length using gaps to account for events of DNA insertion and deletion. The goal is to posit all homologous bases in the sequences in one column that induces a *character* over the set of all possible states. The most prevalent character based methods are maximum likelihood (ML) [7] and maximum parsimony (MP) [10]. ML is model based and attempts to optimize the model parameters to maximize the likelihood, while MP is parameter free and seeks for a tree minimizing the sum of mutations. Although both steps of character based methods, the multiple alignment and the phylogenetic reconstruction, are very computationally intensive, this approach is considered more accurate and a constant effort is being done to improve their performance (e.g., RAxML [22, 23], FastTree [17] and GARLI [27]).

In contrast to character based methods, distance based methods work on pairs of sequences and therefore alleviate the computational burden of considering all sequences simultaneously. Hence, these methods are considered less accurate. Their major advantage is their efficient algorithm and hence its popularity among practitioners. At the first stage, pairwise distances between all pairs of sequences are estimated, based on a specific model of

sequence evolution [12]. This results in a $n \times n$ distance matrix $[D]_{i,j}$. At the second stage, some recursive "cherry-picking" algorithm is applied on the matrix D, resulting in a tree T where the distances between leaves in T, approximate the distances in D. The most popular algorithm for the latter task is neighbor-joining (NJ) [21] that is implemented by a host of software (see e.g. [11, 8, 25])

Sequence based reconstruction is fundamentally based on deep statistical, mathematical and computational foundations with aspect of statistical consistency, fast convergence and computational complexity (see e.g. [1, 2, 14, 5, 6, 13, 15]). These aspects are beyond the scope of the current work (but see the Conclusions Section for further discussion on the subject).

2 Bootstrap

Testing the significance or probability of the resulted phylogeny can be carried out by decay index [4], minimum evolution [19, 20], bootstrap [9] or the comparison of tree Log likelihoods [16] methods. Out of the mentioned methods, the most commonly used is bootstrap analysis. Bootstrap is a statistical method for estimating the distribution of a statistic test by resampling one's data. Normally, in sequence based reconstruction, each site of the aligned sequences is chosen uniformly at random and this procedure is repeated as the length of the aligned sequences.

In order to perform bootstrap analysis on the SI tree a different bootstrap based calculation is required since the SI tree is based on synteny index and not on a set of homologous aligned sequences. Therefore we devised the following approach to allow bootstrap for the new method: for every pair of genomes G_i, G_j from the genome set $\mathcal{G}$, we constructed the SI probability density function $f(SI)$, where, for $0 \leq x \leq 2k$, $f(x)$ holds the number of genes in the union set of genes $G_i \cup G_j$ with $SI = x$. Next we conducted a weighted sampling from that distribution with number of samples $|G_i \cup G_j|$. For example, suppose that a fraction of 0.2 of all the genes in $G_i \cup G_j$ have SI 5. Then the SI value 5 is chosen with probability 0.2. When we average the obtained results of all $|G_i \cup G_j|$ samples, we obtain the bootstrap SI value for genomes G_i, G_j. This value is put in the G_i, G_j entry in the bootstrap distance matrix. Having done so for all pairs, we obtain the *bootstrap SI matrix* from which we build the tree.

The process is iterated as the number of bootstrap iterations defined. We used bootstrap value of 500.

3 Tree Reconstruction Comparisons

To test genome reconstruction methods we first generate random yule tree [26], representing the species evolution, and subsequently evolve an ancestral genome according to it, yielding descendants (taxa) genomes at the leaves. Thereafter we attempt to reconstruct the original tree from the generated taxa genomes. The final step is to compare the original model, species tree and the reconstructed one using the standard Robinson and Foulds (RF) tree comparison measure implemented in the Phylip phylogenetics suit [8].

Pseudo algorithm

1. orig_yule_tree←generate_yule_tree(parameters)
2. taxa_genomes←simulate_taxa_genomes(orig_yule_tree, ancestor_genome)
3. reconstructed_tree←reconstruction_method(taxa_genomes)
4. score ← RF(orig_yule_tree, reconstructed_tree)

3.1 Yule tree generation

A Yule process advances recursively and builds a tree while advancing. At every recursion step the node with the earliest time point is chosen and processed. At node's processing two *edge lengths* are tossed randomly from a predefined distribution. These will be the edge lengths to the two children of the chosen node and their time points will be their ancestor's time plus their edge lengths. When the set of yet unprocessed nodes contains n nodes, the recursion terminates and all nodes are assigned with the time of the earliest unprocessed node. Such a procedure generates an ultrametric tree (or *molecular clock* tree) in which the distance (path length) from the root to any leaf, is the same.
Edge length desceribe a birth Poisson process that distribute exponentially with rate ℓ. p represents the probability of an event occuring during this time period. The procedure receives p as a parameter and transforms it to the corresponding time period, that we denote by *length* $\ell = -log(1-p)$. Next, edge length leading node v is drwan randomly end exponentially, $l_v \sim Exp(\ell)$. Hence we obtain that edge lengths for our tree are exponentially distributed with length $\ell = -log(1-p)$.

3.2 Simulating Genome Evolution

To generate genomes, i.e. gene sequences, according to the species tree we first define the ancestral root genome (usually 1,2,3..N for simplicity) and then propagate it down along the Species tree. Hence, given any ancestral genome we obtain its two children according to their edge lengths with the following procedure - every gene in the child node v's genome undergoes an *event* with probability $1 - e^{-\ell_v}$ where ℓ_v is the length of the node entering v (i.e. the edge from v's ancestor to v). An event can be either an HGT or a gene loss. In a HGT event, a new location on the genome is chosen uniformly and the gene is moved to this location. Otherwise (a gene loss event) the gene is deleted from the genome. In models where no gene loss is permitted (the simulation to genome rearrangement software) all events are HGT. In other models (simulation to gene content or directed pairs) the type of event is determined with probability $\Pr(pHGT)$ (see sub section of simulations for Whole Genome based Reconstruction). Hence, the closer the children to their ancestor, they carry more resemblance to it (in terms of SI), as they underwent less events. This process is repeated recursively along the tree until the leaves are reached. Their resulted genomes are our input taxa genomes which is fed to the reconstruction methods. The procedure also supports more general and more parametrized genomic mutation mechanisms including gene deletion, gene gain, gene block HGT and non uniform HGT new location choice.

Simplified pseudo algorithm:

```
define Simulate genomes(genome, treenode)
    If leaf then
        taxa_genomes ← genome
    Else
        child_1 genome ← mutate genome( father_genome,
                                        mutation_rate=child1_edge_length )
        Simulate_genome(child1_genome, child1)
        child_2 genome ← mutate genome( father_genome,
                                        mutation_rate=child2_edge_length )
        Simulate_genome(child2_genome, child2)

run Simulate genomes(source_genome, source_treenode)
```

3.3 Tree Similarity Measures

There are several approaches to measure similarity between phylogenies. These are normally used in simulation studies where the "true" model tree is known and the accuracy of the reconstruction method is measured by the distance of the reconstructed tree to the model tree. There are several tree metrics. We chose the most common ones:

1. *Robinson-Foulds Symmetric Difference*: The removal of an edge in a phylogenetic tree induces two sub-trees and hence partitions (or splits) the taxa set into two parts. An edge is shared by two trees over the same taxa set, if it induces the same partition in the two trees. The Robinson-Foulds (RF) [18] symmetric difference between two trees is the number of edges (splits) in *exactly* one tree. This is commonly normalized by the total sum of internal edges in both trees to give a number between zero and one. As we here measure similarity rather than distance between trees, we subtract the symmetric difference from one. RF was produced by the function *treedist* in Phylip [8].

2. *Maximum Agreement Subtree (MAST)*: Let T be a tree over a taxa set S and $A \subseteq S$. We denote by $T|_A$ the tree induced by the subset A of leaves, so that all degree-two nodes are suppressed. We also normalize the scores to [0..1] scale for readability and simplicity. The MAST score between two trees T_1, T_2 is the size of the largest subset A, such that $T_1|_A = T_2|_A$. To produce the MAST distance between T_1 and T_2, we normalize the MAST score by the number of leaves common to T_1 and T_2 Note that if the MAST distance between two trees is 1, then the two trees are identical. MAST is implemented by the function *agree* in PAUP* [24].

3. *Quartet Fit*: A *quartet* is a tree on four leaves $\{a, b, c, d\}$. We write a quartet q over $\{a, b, c, d\}$ as $ab|cd$ if there exists an edge in q splitting a, b from c, d. If the quartet is resolved (i.e., binary), then it has an edge separating two of the leaves from the other two. Hence, each binary quartet on a, b, c, d can be written as one of $ab|cd, ac|bd$, or $ad|bc$. When a tree T on the full taxa set S has an edge separating a, b from c, d, then we say that T induces the quartet $ab|cd$, and that the quartet tree $ab|cd$ is *consistent* (or *agrees*) with T. There are $\binom{n}{4}$ quartets in a tree (note that a quartet does not have to be resolved under a given tree). The *quartet fit* [3] measure counts which of all $\binom{n}{4}$ quartets share the same topology between the two trees. As the

size of the trees prohibits testing all $\binom{n}{4}$ combinations of four taxa, we implemented a randomized version that samples uniformly at random a subset of the full $\binom{n}{4}$ set.

3.4 Simulation Results Graphs

Figures 1 and 2 show simulation results measuring quality of reconstruction as a function of k for various HGT rates.

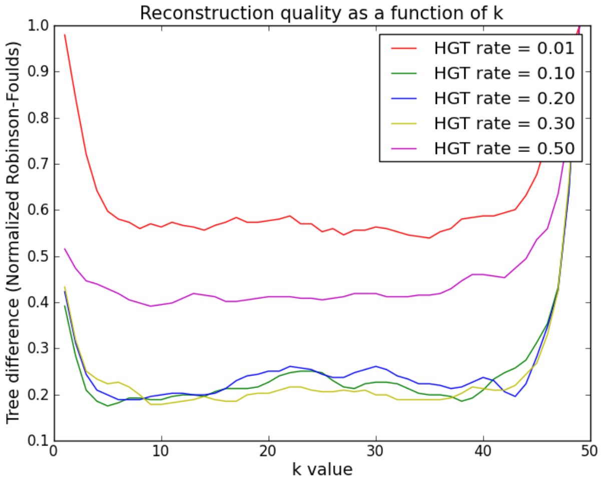

Figure 1: Quality of reconstruction (RF symmetric difference to the model tree) as a function of k for various HGT rate (HGT probability at each a gene in a genome). Simulated number of taxa (n) is 100, genome size is 500.

Figure 2 shows a similar result, only that here k is held constant and the rate of HGT is varied (that is, trees of different lengths were generated). Again, the superiority of small k, but not too small, is shown over too large k's.

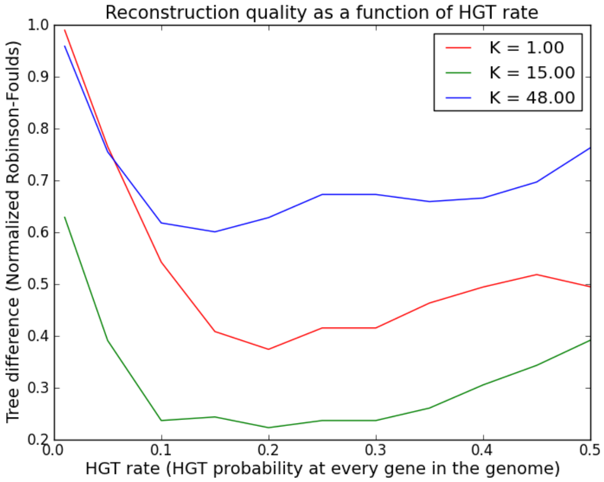

Figure 2: Quality of reconstruction (RF symmetric difference) as a function of HGT intensity for various values of k. Simulated number of taxa (n) is 100, genome size is 500.

3.5 Simulation Results with Related Whole Genome based Reconstruction Techniques

Figures 3, 4, and 5 show results on camparison of Synteny Index vs Directed Pairs vs Gene Content. The different figs show results for different values pHGT (0.9, 0.8, 0.7)

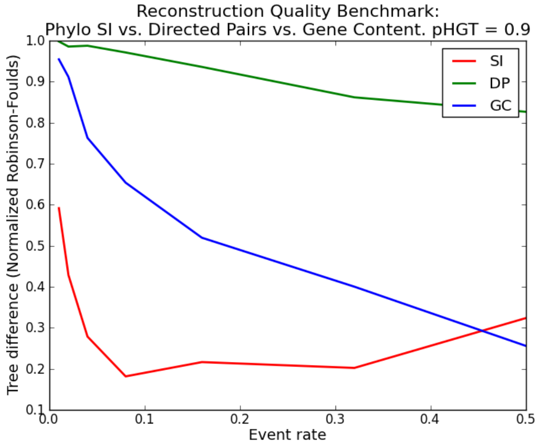

Figure 3: Phylo SI vs. Directed Pairs vs. Gene Content reconstruction quality Benchmark. Measured: reconstruction quality (normalized Robinson-Folds between model and reconstructed trees) as a function of event rate. Probability of event at a gene in a genome distributed exponentially with parameter "event rate". An event at a gene is HGT with probability (pHGT) or gene loss with probability (1-pHGT). Genomes size is 700, number of taxa in the phylogenetic tree is 80, K value in SI method is 10, pHGT (HGT probability at the mutation event in tree simulation) is 0.9.

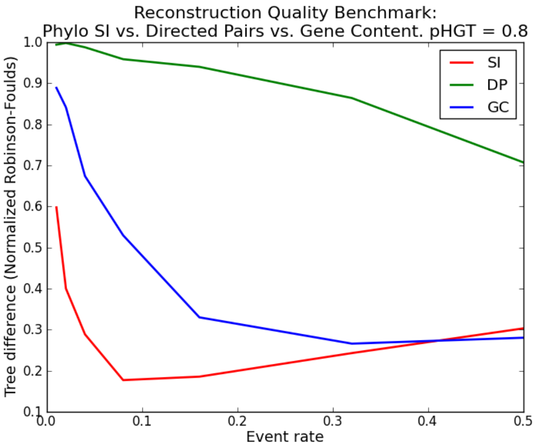

Figure 4: Same parameters as in Figure 3 but pHGT 0.8.

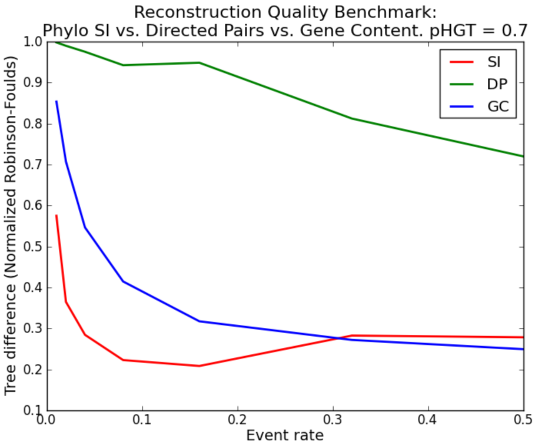

Figure 5: Same parameters as in Figure 3 but pHGT 0.7.

4 The 89 Uniformly Selected Bacterial Trees

4.1 Genome Preprocessing

As described in the main text, we created gene lists, based on RefSeq annotation. RefSeq however contains many spurious entries such as "hypothetical gene" and alike. We therefore applied the following rule. We removed all gene names of length greater than five letters. The average percentage of removed genes was 5.63% (std 0.72) and the average genome length (#genes) after removal was 1782.57. This relatively low percentage shows that the influence of these spurious genes is negligible. Figures 6 and 7 show a histogram of the gene name lengths in the genome analyzed and a histogram of the percentage of genes that were removed in each genome.

Finally, Figure 8 shows a histogram of the genome length after name filtration.

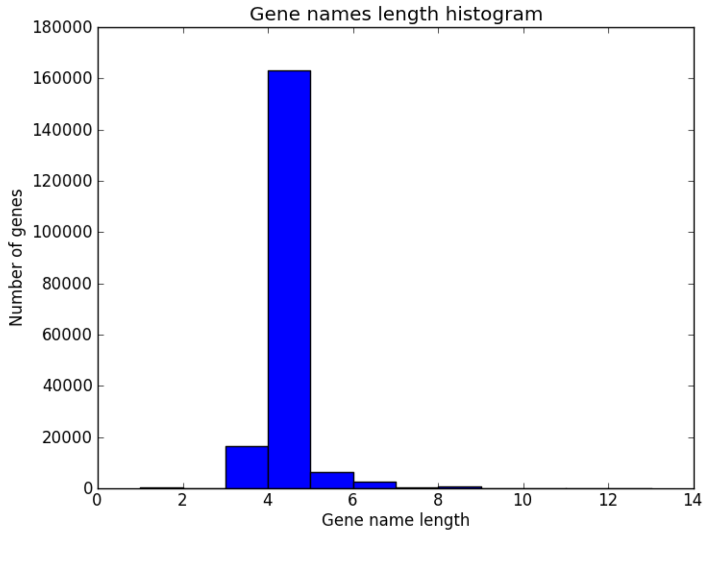

Figure 6: distribution of gene name lengths in the organisms analyzed.

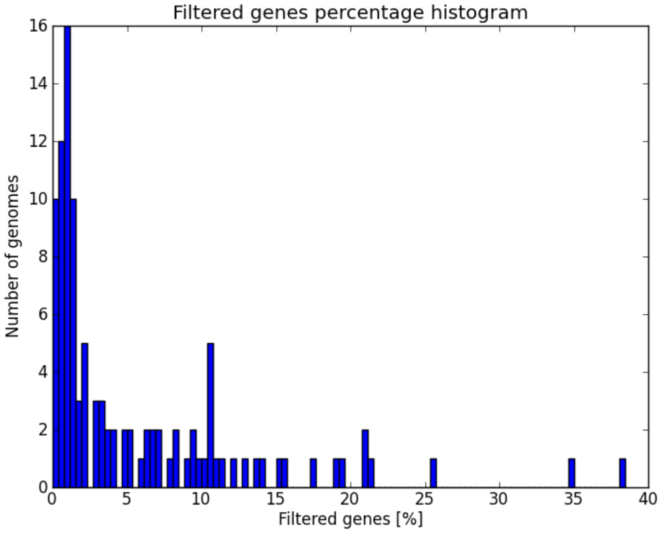

Figure 7: Percentage of genes filtered out from the genomes.

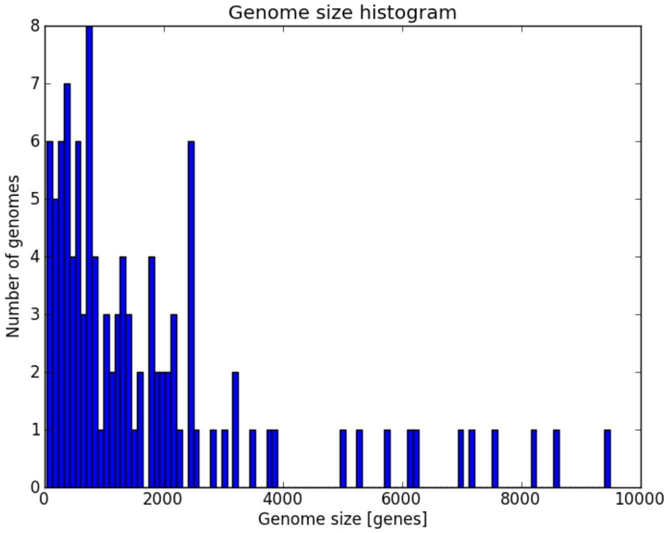

Figure 8: Genome size (#genes) histogram for the genomes analyzed.

4.2 Resulted Trees

We here show the resulted trees by the four methods, the SI-tree, the TOL-tree, the 16s-tree, and the AMPHORA tree. Trees were all rooted identically with *Bacteroides fragilis* as outgroup to facilitate comparison.

The supplementary material contains the trees in Newick format and additionally the original RDP NJ tree and the gene content and directed pairs trees.

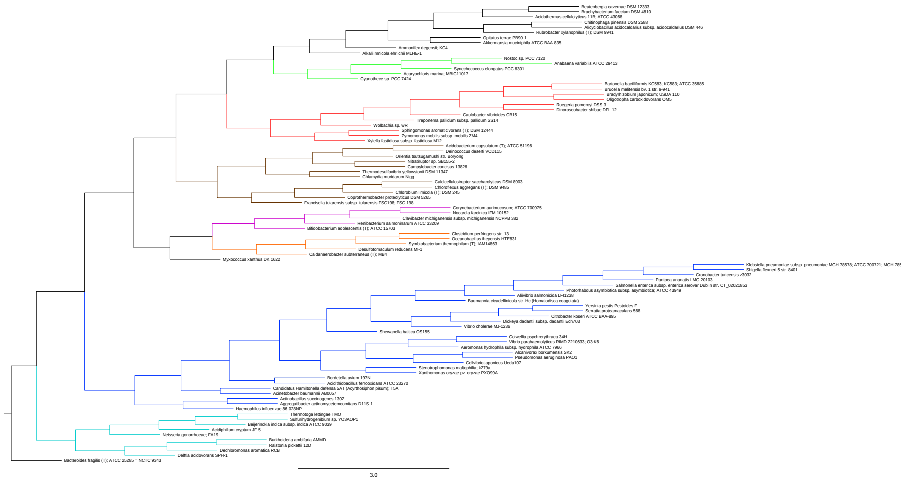

Figure 9: The SI tree on 89 microbial organisms.

The tree was constructed by Neighbor Joining from pairwise distances $[D]_{i,j} = 1 - \overline{SI_{10}}(G_i, G_j)$. The tree is, by construction, fully resolved (86 internal branches).

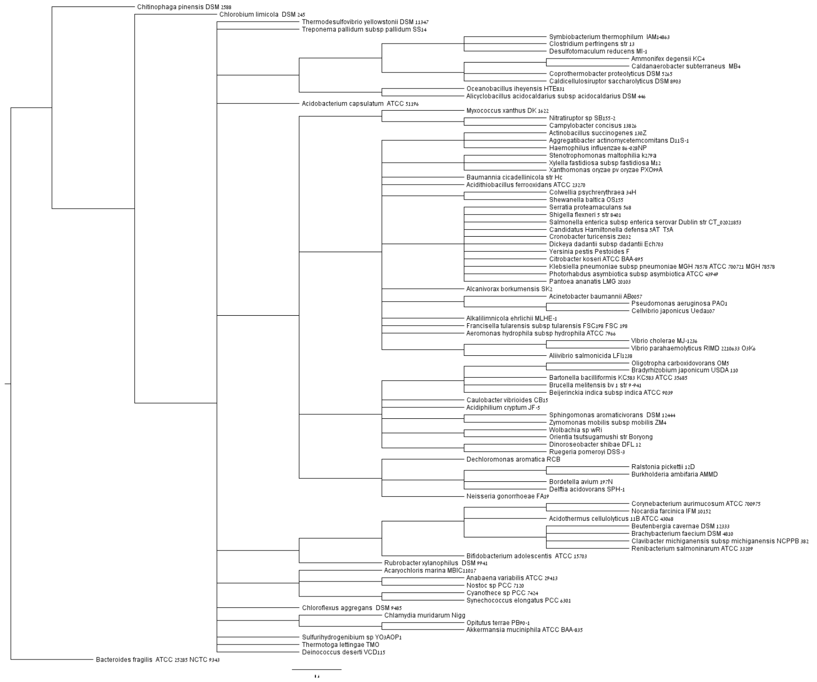

Figure 10: The Tree of Life was constructed using iTOL on 89 microbial organisms. As can be seen the tree is very loosely resolved with only 41 internal branches.

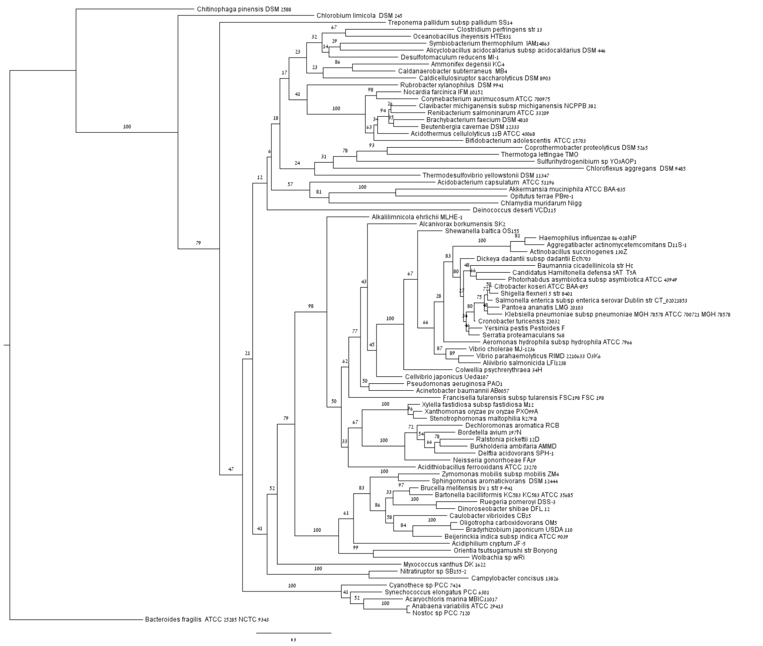

Figure 11: The 16s rRNA tree built by maximum likelihood using PhyML GTR + gamma (designed for sequences with significant between-site rate heterogeneity) over aligned sequences extracted from the Ribosomal Database Project.

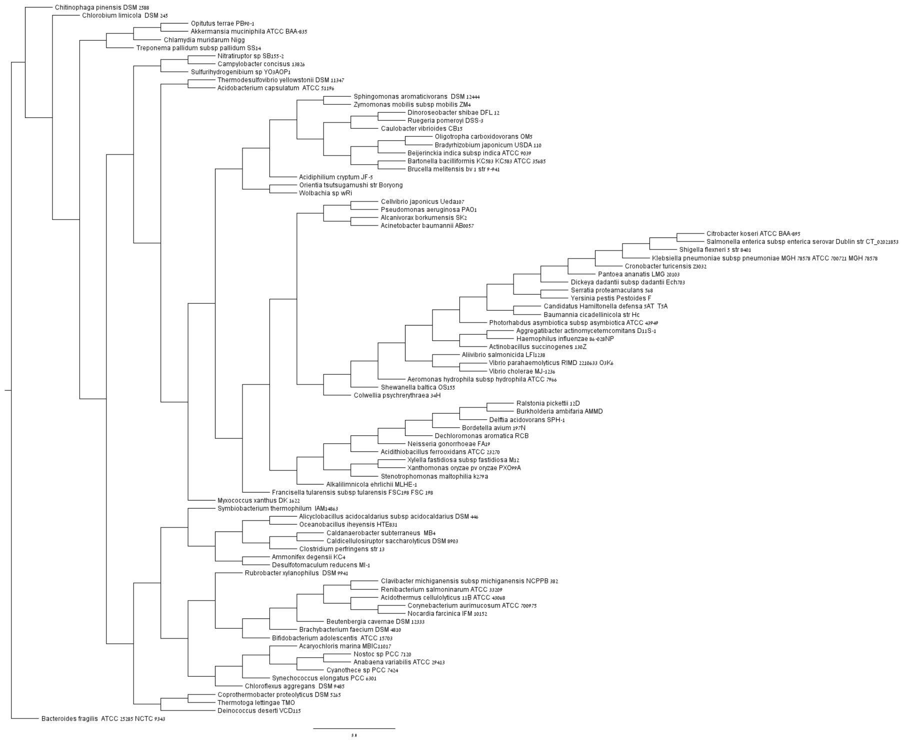

Figure 12: The Amphora tree.

5 The Tree Over Large Enough Genomes

5.1 Genome Size Confidence Values under RefSeq

As was discussed before, RefSeq annotation for several genomes is very sparse, to the degree that some genomes in our 89 uniformly selected genomes, contain only a few dozens of annotated genes, preventing any reliable phylogenetic inference. As it was shown above, the major factor of confidence, is the "coverage" of the genome, that is, the number of identified genes. For this purpose, we devised the following confidence criterion. Let $g_i = |G_i|$ be the "size" of genome i in terms of the number of annotated genes under RefSeq, and g_M be the maximum g_i over all genomes in the set. Then, for a leaf G_i in the tree, the confidence of the branch emanating from G_i, C_i, is defined as $C_i = \log g_i / \log g_M$. Alternatively, for an internal node i, let t_i be the tree (clade) rooted under it, and $|t_i|$ the number of leaves in t_i. Then, the confidence of internal node i is a weighted sum of the confidence of its children where the weight of a child j is its subtree size $|t_j|$ and the total sum is normalized by the subtree size under node i, $|t_i|$. It is easy to see that C_i is at most 1 and equals 1 only for the genome with the maximum number of annotated genes.

5.2 The 500+ Genes Genome Tree

We filtered out all genomes with less than 500 genes and left with only 47 organisms, where the maximally annotated genome is of *Pantoea ananatis* with 3494 genes. The table, with confidence values is found in the supplementary material. The resulted tree is shown in Figure 13

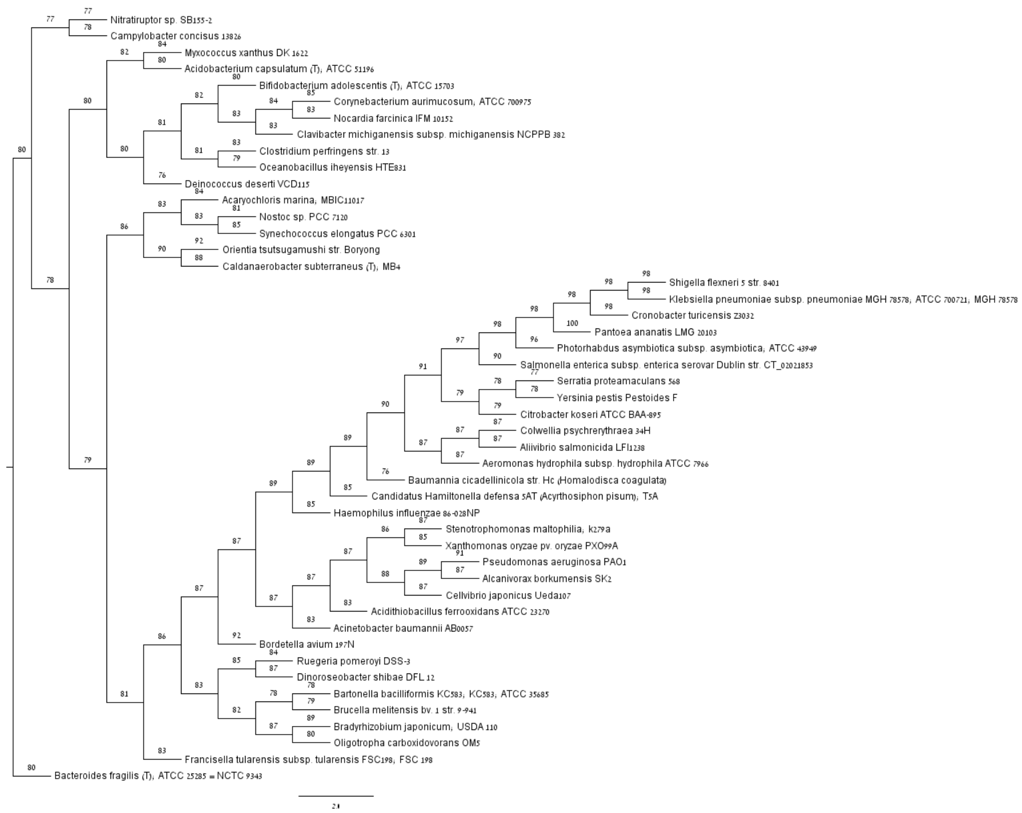

Figure 13: The tree over genomes of at least 500 genes. The numbers at the nodes represent the confidence values for the respective nodes.

6 The Alphaproteobacteria Trees

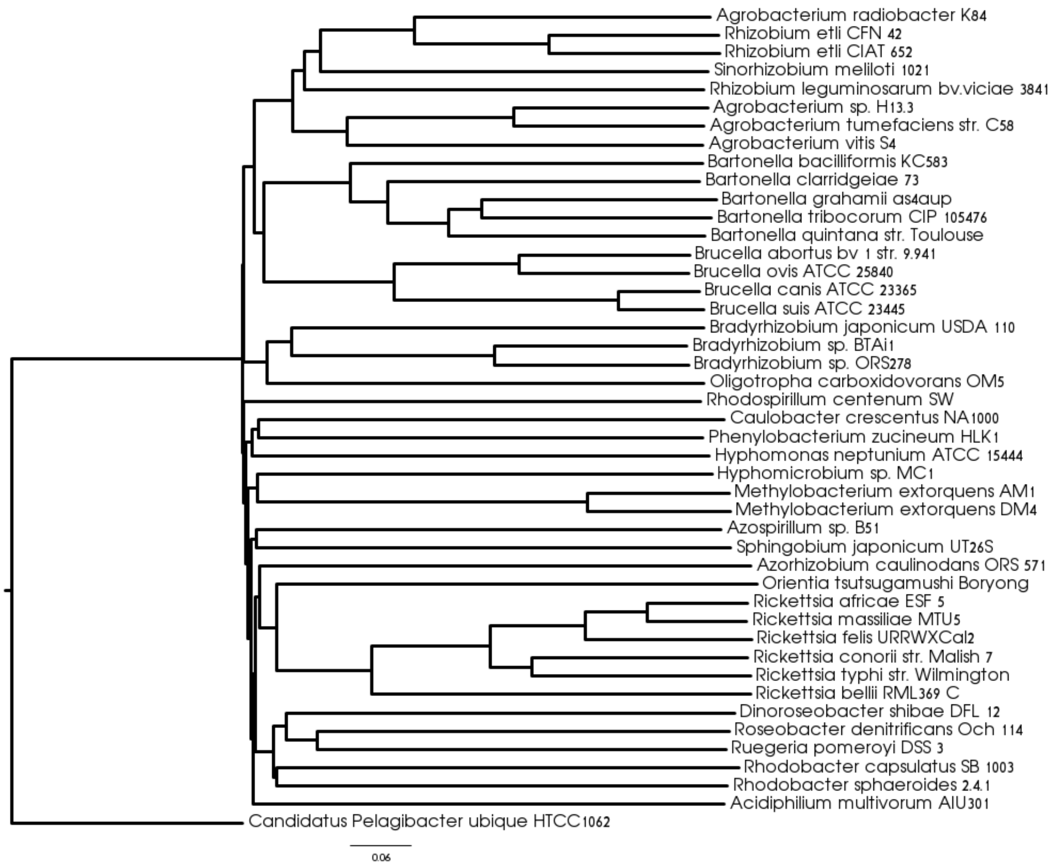

Figure 14: The SI tree on 45 α-*proteo bacteria* organisms. The tree was constructed by Neighbor Joining from pairwise distances $[D]_{i,j} = 1 - \overline{SI}_{10}(G_i, G_j)$.

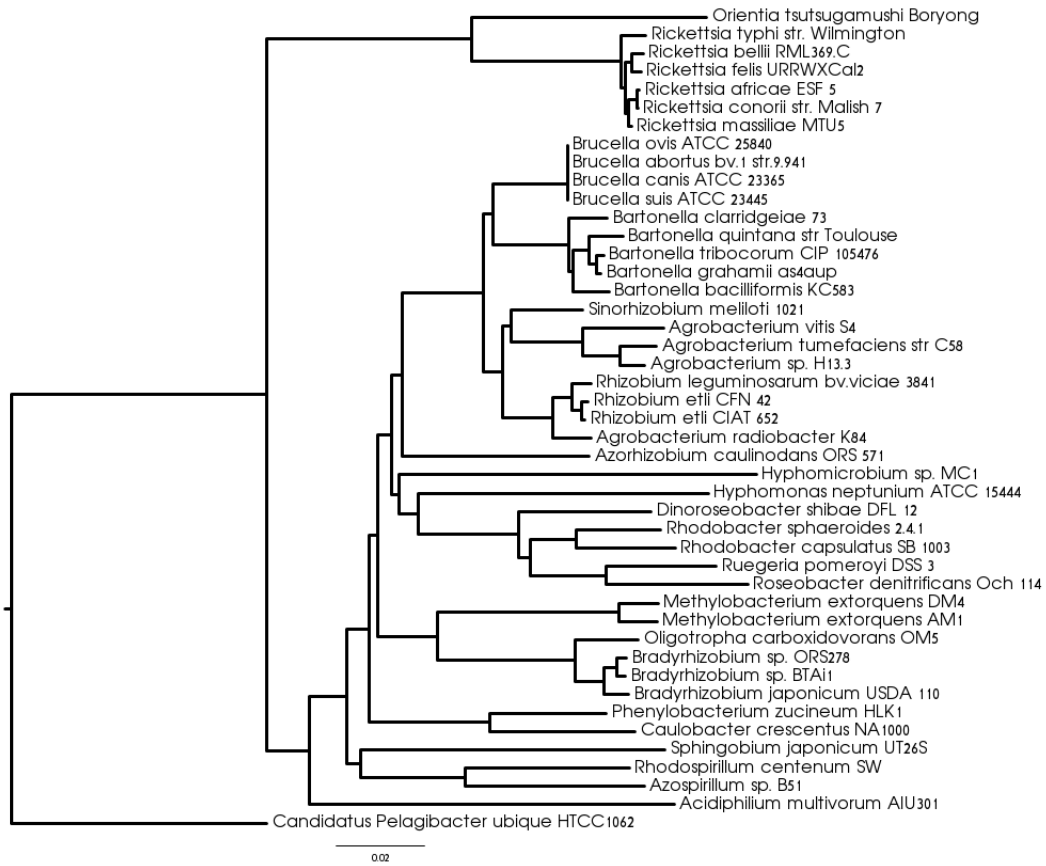

Figure 15: The 16s rRNA tree extracted from the Ribosomal Database Project.

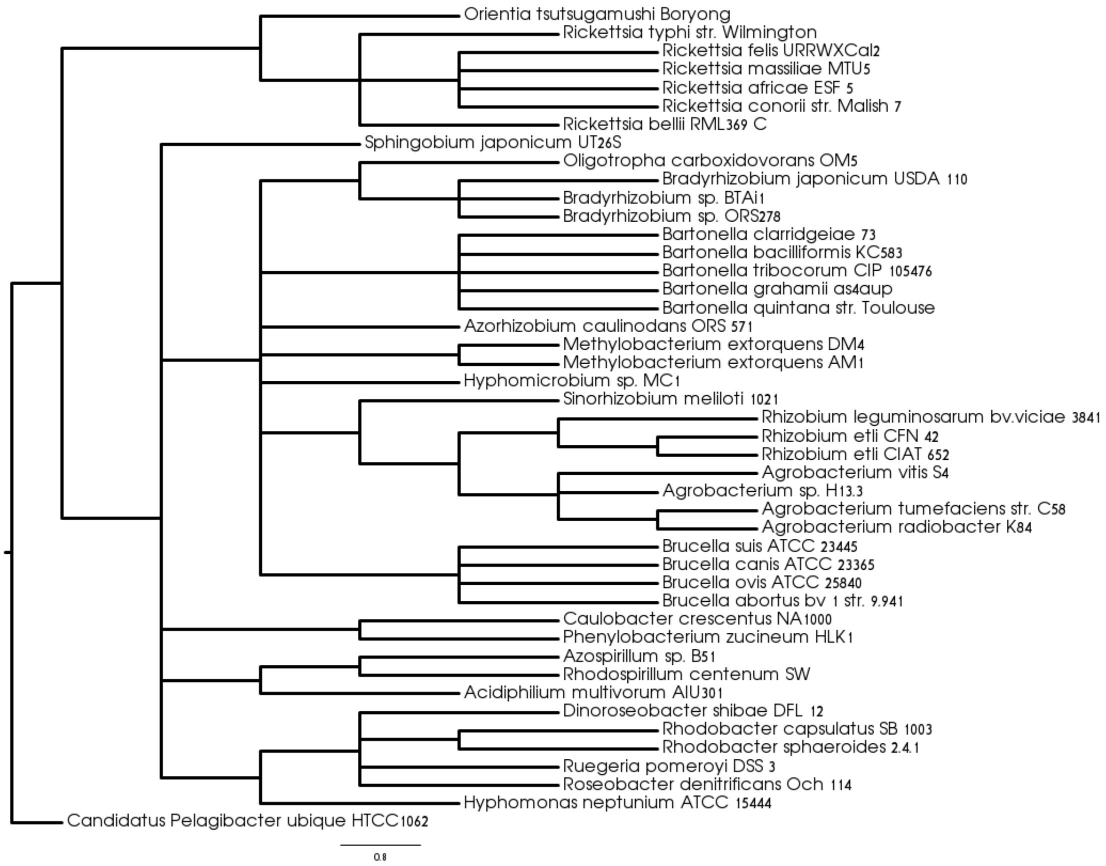

Figure 16: The Tree of Life built using iTOL

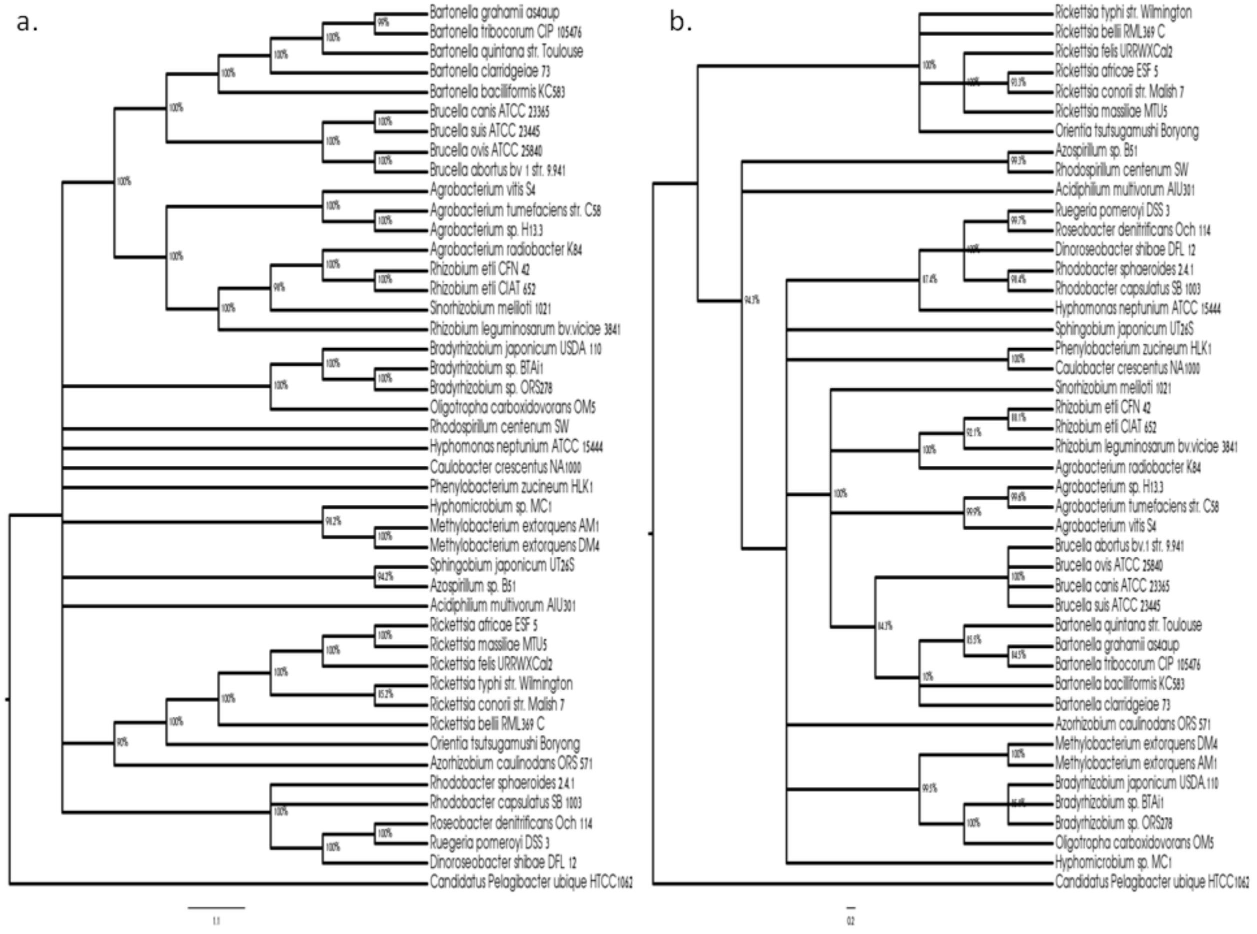

Figure 17: The bootstrap trees contain only edges with more the 80% values a. 16S bootstrap tree b. SI bootstrap tree

References

[1] Constantinos Daskalakis, Cameron Hill, Alexander Jaffe, Radu Mihaescu, Elchanan Mossel, and Satish Rao. Maximal accurate forests from distance matrices. In *RECOMB*, pages 281–295, 2006.

[2] Constantinos Daskalakis, Elchanan Mossel, and Sébastien Roch. Optimal phylogenetic reconstruction. In *STOC*, pages 159–168, 2006.

[3] William H. E. Day. Analysis of quartet dissimilarity measures between undirected phylogenetic trees. *Systematic Zoology*, 35(3):pp. 325–333, 1986.

[4] Michael J. Donoghue, Richard G. Olmstead, James F. Smith, and Jeffrey D. Palmer. Phylogenetic relationships of dipsacales based on rbcl sequences. *Annals of the Missouri Botanical Garden*, 79(2):pp. 333–345, 1992.

[5] P. Erdös, M. Steel, L. Szekely, and T. Warnow. A few logs suffice to build (almost) all trees (i). *Random Structures and Algorithms*, 14:153–184, 1999.

[6] P. Erdös, M. Steel, L. Szekely, and T. Warnow. A few logs suffice to build (almost) all trees (ii). *Theoretical Computer Science*, 221:77–118, 1999.

[7] J. Felsenstein. Evolutionary trees from dna sequences: a maximum likelihood approach. . *J Mol Evol.*, 17(6):368–76, 1981.

[8] J. Felsenstein. PHYLIP - phylogenetic inference package, (version 3.2). *Cladistics*, 5:164–166, 1989.

[9] Joseph Felsenstein. Confidence limits on phylogenies: An approach using the bootstrap. *Evolution*, 39(4):pp. 783–791, 1985.

[10] W. Fitch. Toward defining the course of evolution: minimum change for a specified tree topology. *Syst. Zool.*, 20:406–416, 1971.

[11] O. Gascuel. BIONJ: An improved version of the NJ algorithm based on a simple model of sequence data. *MBE*, 14(7):685, 1997.

[12] D. Graur and W. Li. *Fundamentals of Molecular Evolution.* Sinauer Assoc., 2000.

[13] I. Gronau, S. Moran, and S. Snir. Fast and reliable reconstruction of phylogenetic trees with very short branches. In *ACM-SIAM Symposium on Discrete Algorithms (SODA)*, pages 379–388, 2008.

[14] M. D. Hendy and D. Penny. Spectral analysis of phylogenetic data. *J. Classif.*, 10:5–24, 1993.

[15] K. St. John, T. Warnow, B. M. E. Moret, and L. Vawter. Performance study of phylogenetic methods: (unweighted) quartet methods and neighbor-joining. In *Proceedings of the Sixth Annual ACM-SIAM Symposium on Discrete Algorithms*, 2001.

[16] Hirohisa Kishino and Masami Hasegawa. Evaluation of the maximum likelihood estimate of the evolutionary tree topologies from dna sequence data, and the branching order in hominoidea. *Journal of Molecular Evolution*, 29:170–179, 1989. 10.1007/BF02100115.

[17] Morgan N. Price, Paramvir S. Dehal, and Adam P. Arkin. Fasttree 2 approximately maximum-likelihood trees for large alignments. *PLoS ONE*, 5(3):e9490, 03 2010.

[18] D.R. Robinson and L.R Foulds. Comparison of phylogenetic trees. *Mathematical Biosciences*, 53:131–147, 1981.

[19] A. Rzhetsky and M. Nei. A simple method for estimating and testing minimum-evolution trees. *Mol. Biol. Evol*, 9(5):945–967, 1992.

[20] Andrey Rzhetsky and Masatoshi Nei. Metree: a program package for inferring and testing minimum-evolution trees. *Computer applications in the biosciences : CABIOS*, 10(4):409–412, 1994.

[21] N. Saitou and M. Nei. The neighborjoining. *MBE*, 4:418–427, 1987.

[22] A. Stamatakis. Raxml-vi-hpc: Maximum likelihood-based phylogenetic analyses with thousands of taxa and mixed models. *Bioinformatics*, 22::2688–2690, 2006.

[23] Alexandros Stamatakis, Paul Hoover, and Jacques Rougemont. A rapid bootstrap algorithm for the raxml web servers. *Systematic Biology*, 57(5):758–771, 2008.

[24] D.L. Swofford. *PAUP*beta*, 1998. Sinauer, Sunderland, Mass.

[25] K. Tamura, D. Peterson, N. Peterson, G. Stecher, M. Nei, and S. Kumar. Mega5: Molecular evolutionary genetics analysis using maximum likelihood, evolutionary distance, and maximum parsimony methods. *MBE*, 28(10):2731–2739, 2011.

[26] G. Udny Yule. A mathematical theory of evolution, based on the conclusions of dr. j. c. willis, f.r.s. *Philosophical Transactions of the Royal Society of London. Series B, Containing Papers of a Biological Character*, 213, 1925.

[27] D. J. Zwickl. *Genetic algorithm approaches for the phylogenetic analysis of large biological sequence datasets under the maximum likelihood criterion.* PhD thesis, The University of Texas at Austin, Austin, USA, 2006.
